# Qing-Kai-Ling oral liquid alleviates non-alcoholic fatty liver disease via remodeling gut microbiota and activating AMPK/ACC1 axis

**DOI:** 10.1186/s13020-025-01237-4

**Published:** 2025-10-19

**Authors:** Kaiwei Cai, Zihao Chen, Jingyun Wu, Qiuyun Wang, Xiaoqin Zhou, Biyan Pan, Zhiyong Xie, Pei Li, Fenglian Chen, Hongying Chen, Qiongfeng Liao

**Affiliations:** 1 State Key Laboratory of Traditional Chinese Medicine Syndrome, School of Pharmaceutical Sciences, Guangzhou University of Chinese Medicine, 232 Waihuan East Road, University Town, Panyu District, Guangzhou, 510006 Guangdong People’s Republic of China; 2 Guangzhou Baiyunshan Mingxing Pharmaceutical Co.,Ltd., No.52, Xinshi Street Xiaogang Dama Road, Guangzhou, 510250 Guangdong People’s Republic of China; 3 School of Pharmaceutical Sciences (Shenzhen), Sun Yat-Sen University, Guangzhou, 510006 Guangdong People’s Republic of China

**Keywords:** Qing-Kai-Ling (QKL) oral liquid, Non-alcoholic fatty liver disease, Gut microbiota, AMPK/ACC1 pathway, Short-chain fatty acids

## Abstract

**Background:**

Qing-Kai-Ling (QKL) oral liquid, evolving from a classical Chinese formula known as An-Gong-Niu-Huang pills, has demonstrated hepatoprotective, lung-protective, and gut microbiota-modulating properties. However, its efficacy in preventing high fat diet (HFD)-induced non-alcoholic fatty liver disease (NAFLD) and its relationship with gut microbiota and hepatic inflammation remain unclear.

**Purpose:**

The study aims to investigate whether QKL can prevent HFD-induced NAFLD, focusing on the mechanistic role of gut microbiota, microbial metabolites, and hepatic inflammation.

**Methods:**

QKL was subjected to extraction and chemical profiling to identify its active compounds. In vivo studies were conducted in HFD-fed mice to assess the effects of QKL on hepatic lipid accumulation, inflammation, gut microbiota composition, SCFAs production, intestinal permeability, body weight, and fat mass.

**Results:**

Chemical analysis revealed that the major components of QKL are gallic acid, corilagin, and chebulagic acid. QKL administration (12.33 and 24.66 mL/kg) for 8 weeks significantly reduced hepatic steatosis, serum lipid profiles (TG, LDL-C), and body weight in high-fat diet-induced NAFLD mice, while improving glucose tolerance and intestinal barrier integrity. Gut microbiota analysis revealed QKL enriched beneficial taxa (e.g., *Akkermansia*, *Bacteroides*) and suppressed pathobionts (e.g., *Lachnospiraceae NK4A136_group*), effects replicated through faecal microbiota transplantation from QKL-treated donors. QKL upregulated intestinal gene *GPR41/43* and hepatic protein GPR135 expression, enhanced SCFAs production (acetic, propionic, and butyric acids), and activated AMPK/ACC1 signaling to suppress lipogenesis and promote lipid oxidation. Untargeted metabolomics demonstrated QKL restored hepatic fatty acid metabolism by reducing palmitic acid and arachidonic acid accumulation.

**Conclusion:**

These findings established QKL as a microbiota-modulating therapeutic agent for NAFLD through SCFA-AMPK/ACC1 axis activation, providing a foundation for developing QKL-based treatments.

**Graphical Abstract:**

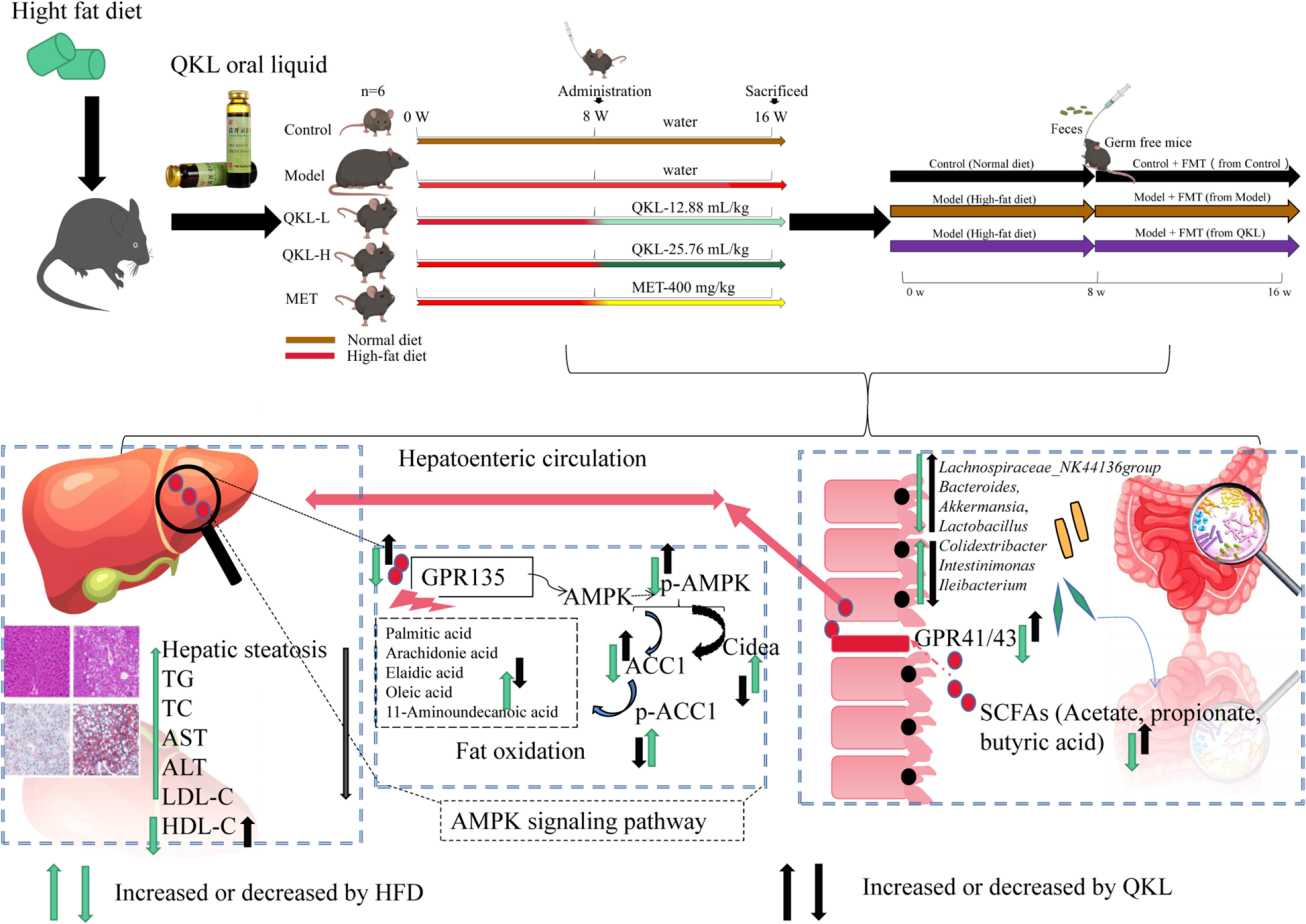

**Supplementary Information:**

The online version contains supplementary material available at 10.1186/s13020-025-01237-4.

## Introduction

Non-alcoholic fatty liver disease (NAFLD) was a pathogenic and clinical condition described by an overaccumulation of fat in the cells of the liver. The worldwide occurrence of NAFLD rose to 34% and was expected to attain 56% by the year 2030, positioning it as a primary contributor to chronic liver disease and hepatocellular cancer [1]. The pathogenesis of NAFLD was complex, involving various metabolic and inflammatory pathways, and specific drugs remained lacking. Research indicated a strong connection between NAFLD onset and gut microbiota dysbiosis [2, 3]. Furthermore, faecal microbiota transplantation (FMT) enhanced NAFLD conditions via altering the microbiota composition in the gut [4, 5]. Consequently, focusing on the targeted modulation of the gut microbiota had come forth as a potential therapeutic approach for treating NAFLD.

The microbiota in the gut, specifically a decline in the Firmicutes to Bacteroidetes ratio (F/B), had been recognised as a vital element in improving NAFLD [6]. Both Firmicutes and Bacteroidetes contributed to the dietary fibre fermentation process into short-chain fatty acids (SCFAs), such as acetate, propionate, and butyrate [7, 8]. SCFAs served crucial physiological regulatory functions by supplying energy, balancing lipid metabolism, safeguarding the barrier of the intestine, enhancing nutrient absorption, and suppressing fat accumulation [9, 10]. Several investigations had proved that individuals with NAFLD often displayed a modified gut microbiota profile, leading to decreased SCFA production [11, 12]. New research had underscored the intertwined functions of G protein-coupled receptor 41/43 (GPR41/43), SCFA receptors implicated in energy homeostasis, with its activation boosting adenosine 5′-monophosphate (AMP)-activated protein kinase (AMPK) phosphorylation in hepatocytes [13]. Although there was no direct evidence confirming SCFA-mediated activation of GPR135, the metabolic and inflammatory processes regulated by SCFAs through other receptors (e.g., FFAR2/3) were suggested to synergise with GPR135’s protective effects in NAFLD [14].

The activation of AMPK has been demonstrated to phosphorylate and hinder acetyl-CoA carboxylase 1 (ACC1), which was an enzyme that limited rates in the process of fatty acid synthesis, thereby reducing malonyl-CoA production and promoting mitochondrial β-oxidation [15]. Concurrently, AMPK activation suppressed cell death-inducing DFFA-like effector A (CIDEA) protein, which stabilised lipid droplets and facilitated lipid storage [16]. In NAFLD, axis dysfunction is marked by impaired GPR135 signalling, AMPK pathway inactivation, hyperactivation of ACC1, and pathological upregulation of CIDEA, which synergistically promotes hepatic lipid deposition and exacerbates insulin resistance [17]. These interactions collectively formed a coordinated mechanism to maintain energy and lipid homeostasis. This evidence underscored the critical role of SCFAs in fat metabolism and their impact on metabolic diseases like obesity by modulating the AMPK/ACC1 signalling pathway.

Qing-Kai-Ling (QKL), a traditional Chinese herbal formula derived from the Angong Niuhuang pill, combined bioactive components such as Margaritifera Concha, Gardenia jasminoides, and cholic acids, demonstrating potent anti-inflammatory and antioxidant effects [18]. Emerging evidence suggested that QKL ameliorated metabolic syndrome-related conditions, including liver injury, by modulating pro-inflammatory cytokines and mitigating redox imbalance [19, 20]. Contemporary research highlighted its capacity to reshape gut microbiota composition in pneumonia models, fostering the enrichment of beneficial taxa (e.g., *Lactobacillus*) and suppressing pathogenic bacteria [18]. Given the pivotal role of gut microbiota-derived SCFAs in regulating hepatic lipid metabolism via the AMPK/ACC1 axis, a pathway critical for fatty acid oxidation and lipid droplet stabilisation, we hypothesised that QKL alleviated NAFLD by restoring microbial homeostasis and enhancing SCFA-mediated signaling. It was known that SCFAs (like acetate and propionate) could increase AMPK activation, stop ACC1 from making fat, and stop CIDEA from keeping fat in the body [21, 22]. But no one had looked into how QKL, gut bacteria, and this metabolic pathway interact in NAFLD. Specifically, it was unclear whether QKL’s anti-steatotic effects depended on its ability to elevate SCFA levels, thereby activating GPR135 to suppress hepatic ACC1 and CIDEA expression. To address this gap, we investigated QKL’s therapeutic efficacy in a high-fat diet (HFD)-induced NAFLD murine model, focusing on its dual regulation of gut microbiota-SCFA crosstalk and downstream lipid metabolic reprogramming.

In this research, we utilised a mouse model subjected to an HFD to illustrate the defensive effect of QKL in combating NAFLD, which operates via the modulation of microbiome in the gut and SCFA metabolism, thereby influencing the AMPK/CIDEA signaling pathway. The objective of this research was to provide fresh perspectives on the preventative impacts of QKL concerning NAFLD by examining its interactions with gut microbiota and metabolic byproducts.

## Materials and methods

### Materials

QKL was procured from Guangzhou Baiyunshan Mingxing Pharmaceutical Co., Ltd., located in Guangzhou, China. Additionally, Metformin (MET), which served as the reference drug for our experiments, was acquired from Sigma-Aldrich, based in St. Louis, Germany. The HFD utilised in our research, specifically D12492, contained 60% fat, 20% protein, and 20% carbohydrates and was sourced from Research Diets Co., Inc. For the biochemical analyses, essential testing kits to measure aspartate aminotransferase (AST), alanine aminotransferase (ALT), total cholesterol (TC), triglycerides (TG), high-density lipoprotein cholesterol (HDL-C), and low-density lipoprotein cholesterol (LDL-C) CCK-8 assays were acquired from Jiancheng Bioengineering Institute. Oleic acid and palmitic acid were provided by Macklin. The oil red staining kit was purchased from Solabao. Isoflurane, a key component for the experimental procedures, was acquired from Aibei Biotechnology. The antibiotics were employed from Macklin. Neomycin sulphate was sourced from Bidepharm. Additionally, mouse MUC-2 and occludin-1 were sourced from Abcam, which were crucial for our analysis. Bovine serum albumin (BSA), a protein used in various assays, was obtained from BioFROXX. Difco skim milk, which has applications in blocking agents during testing, was obtained from BD. It is important to note that all supplementary chemicals utilised in the research were of analytical-grade purity or even higher purity, ensuring the integrity and reliability of the experimental results.

### Animal experiment

Guangdong Medical Laboratory Animal Centre provide C57BL/6 J mice [registration number SCXK (YUE) 2024–0202] and kept in regular settings, which included a 12-h light/dark cycle, a temperature range of 22 ± 2 °C, and a humidity of 55 ± 5%. After a week of acclimatization before the experiment, the mice were divided into two groups at random: one that mimicked non-alcoholic fatty liver disease (Model, *n* = 40) and another that served as a control group (Control, *n* = 10). A high-fat diet (HFD) was given to the Model group for 16 weeks in order to create NAFLD, whereas the mice in normal group was given a regular diet and water. After the modeling, at the 8th week, the successful construction of the model could be judged by observing the weight of mice, blood cholesterol, sugar tolerance, insulin resistance, liver fat changes, and corresponding adjustments could be made. After the model was well-established. Besides the Control group, the model group consisted of 40 mice that were randomly assigned into four subgroups (*n* = 10 mice for each subgroup): HFD group, HFD + QKL-H group (24.66 mL/kg), HFD + QKL-L group (12.33 mL/kg), and MET group (400 mg/kg). Following an 8-week modeling period, the mice in the QKL and MET subgroups received intragastric administrations of their respective doses of QKL and MET daily for another 8 weeks. Meanwhile, the same amount of regular saline was administered to the others. Every week throughout the course of the trial, the weights of the mice were noted. At week 16, the body surface fat of mice was measured using QMR12-060H-I small animal MRI (NiuMag, China). Serum and livers were taken after the mice were humanely put to euthanize after the last dose was given. To aid in histological analysis, a portion of the liver tissue that had been taken was submerged in a 4% paraformaldehyde solution. To preserve their integrity for further study and examination, the remaining liver sections were kept at a temperature of − 80 °C. The Guangzhou University of Chinese Medicine’s Animal Ethics Committee authorized all of the study’s animal programs (License No. ZYD2023-231).

### Germ-free mice

A mixture of antibiotics was used to try to drastically lower the amount of bacteria in the gastrointestinal tract of certain pathogen-free mice. The antibiotic cocktail, comprised ampicillin, neomycin, metronidazole at 1 g/L respectively, and vancomycin at 0.5 g/L [5]. These antibiotics were mixed and dissolved in sterile distilled water to reach the specified concentrations, allowing for a comprehensive targeting of both gram-positive and gram-negative bacterial populations. Over a period of 10 days, six mice belonging to the Model group were allowed to consume this antibiotic solution freely. In contrast, to provide a baseline for comparison, the Control group’s mice received the same amount of sterile distilled water over the same period of time. After the 10-day treatment period, the fecal matter from all the mice was collected for analysis. The DNA was extracted from the feces, and agarose gel electrophoresis was employed to assess the effectiveness of the antibiotic regimen in eliminating the intestinal bacteria of the host. Detailed procedures regarding the horizontal gel electrophoresis method could be found in Supplementary Method 1.

### Fecal microbiota transplantation to germ-free mice

The Model group was given a HFD for 8 weeks, while the Control group was maintained on a standard chow. These mice were then randomly assigned to receive fecal samples from either the Control group or the QKL-treated conventional group (*n* = 10 per group) every day. The method of FMT follows previous studies [23]. To guarantee full homogeneity of the sample, 1 g of feces was combined with 5 millilitres of sterile PBS and spun for 5 min. Thereafter, the solution was centrifuged (5 min, 4 °C, 800 g). At week sixteen after treatment, mice were killed. Animal Ethics License No. ZYD-2023-261.

### Sample collection

For FMT studies, mouse feces were collected on the first day after administration of the drugs, and for 16S rRNA and non-target metabolomics analysis, mouse feces were obtained 1 week before the end of the experiment. During the collection procedure, all excrement was promptly kept at − 80 °C. Following a 16-h fast, the mice were anaesthetized by CO_2_ inhalation, and the weight of each group was noted. Blood was obtained at the end of the animal experiment at week 16. The top serum layer centrifuging (3000 rpm, 4 °C, 10 min). The weights of the liver and white adipose tissue were then determined. A 4% paraformaldehyde solution was then used to fix the liver and small intestine.

### Chemical composition identification of QKL

QKL oral liquid (50% methanol-diluted, 0.22 μm filtered) were analyzed using UPLC-Q-TOF/MS (detailed parameters in Supplementary method 2), following chromatographic and mass spectrometric conditions previously established by our group. Data acquisition and compound annotation were performed via MZmine 2 and molecular formula prediction tools as described in our published methodology [26].

### Biochemical index assessment

We used kits provided by Nanjing Jiancheng Bioengineering Institute (Nanjing, China) to assess biochemical indices in mouse serum. Aspartate transaminase (AST), alanine aminotransferase (ALT), low-density lipoprotein cholesterol (LDL-C), high-density lipoprotein cholesterol (HDL-C), total cholesterol (TC), and triglycerides (TG) were among the parameters evaluated.

### Histopathological section experiment

Mice specimens were collected, stored in 4% paraformaldehyde, and then sectioned, dehydrated, embedded, and stained before being prepared for hematoxylin–eosin (H&E) staining. The liver and small intestine histopathologic sections were photographed using a 400 × magnification lamp (Nikon Eclipse Ci, Japan). Liver was initially fixed in 4% paraformaldehyde, promptly transferred to liquid nitrogen for rapid freezing, followed by Oil Red O staining to assess lipid accumulation. Paraffin-embedded sections of the small intestine were subjected to dewaxing and graded dehydration, after which antigen retrieval was performed using EDTA buffer. The sections were incubated with anti-MUC-2 antibody at 4 °C for 8 h. Subsequently, an HRP-conjugated secondary antibody was applied, and chromogenic detection was carried out using DAB solution in combination with hematoxylin counterstaining for 60 min. Images were acquired using a light microscope. Immunofluorescence staining was performed in strict accordance with the manufacturer’s protocol (Beyotime, P0175), and the primary antibody against occludin was sourced from Abcam.

### 16S rRNA gene sequencing

Intestinal specimens were obtained from three experimental groups of mice Control, Model, and QKL-H with six biological replicates per group, yielding a total of 18 samples for 16S rRNA gene sequencing. Fecal microbial DNA was purified using the HiPure Stool DNA Kit according to the standardized procedures. Following amplification and purification, PCR products underwent Illumina HiSeq 2500 sequencing (Gene Denovo Bio-Tech, China) for high-throughput analysis, in accordance with standard high-throughput sequencing protocols. Bioinformatic analyses were conducted via the OmicShare (https://www.omicshare.com). All raw sequencing files were archived in the NCBI SRA repository (accession: PRJNA825784).

### Gas chromatography (GC)-based SCFAs metabolomic analysis

Faecal samples (0.1 g each) were accurately weighed and homogenised. Subsequently, 1 mL of PBS was added, followed by vigorous vortexing to ensure thorough mixing. The mixtures were then centrifuged (12,000 rpm, 10 min), and the resulting supernatants were carefully collected. 2-ethylbutyric acid was mixed with each supernatant for quantitative analysis.

SCFAs were quantified using an Agilent 7890B gas chromatography system coupled with a flame ionisation detector (FID). Chromatographic separation was achieved on an Agilent ADB-FFAP capillary column (30 m × 0.25 mm ID, 0.25 μm film thickness). Operational parameters included injector and detector temperatures of 250 °C and 300 °C, respectively. The thermal gradient protocol comprised an initial hold at 60 °C for 2 min, followed by heating to 140 °C (10 °C/min, 2.5 min retention) and subsequent ramping to 200 °C at an identical rate. Helium served as the carrier gas, with a constant flow rate of 1.0 mL/min. SCFAs were identified by comparing retention times with those of authentic standards. Quantification was based on the peak area ratio relative to the internal standard. Calibration curves were constructed for acetic, propionic, butyric, isobutyric, valeric, and isovaleric acids to ensure accurate and reliable measurement. The detection method of SCFAs follows the previous study [23].

### Transcriptome sequencing and bioinformatics analysis

Metware Company (Wuhan, China) performed the transcriptome sequencing of the entire genome. Colon tissue was processed to extract total RNA utilising TRIzol^®^ reagent (MA, USA). Subsequently, RNA quality control and quantification were conducted with Nanodrop 2000 (SC, USA), along with biowest agarose and the Bioanalyzer 5300 (SC, USA). The preparation of the library and the RNA-seq transcriptome sequencing were carried out using the Illumina^®^ platform (CA, USA). Enrichment profiling of DEGs in GO categories and metabolic pathways was performed against the complete transcriptomic background. Statistical significance for enrichment was determined using Bonferroni correction, with a threshold set at *P* ≤ 0.05. The KEGG mapper tool was employed for pathway topology by KOBAS-i (Beijing, China). Gene target network interaction analysis was conducted with Cytoscape version 3.10.3 (Beijing, China). An intergroup correlation heat map analysis using Pearson’s method was generated with Origin 2020 (MA, USA). Gene expression profiling analysis and splicing analysis were provided in Supplement method 4.

### Untargeted metabolomic analysis based on UPLC-Q/TOF–MS

Untargeted metabolomic profiling was performed on a Waters ACQUITY UPLC I-Class Plus system integrated with a Xevo G2-XS Q-TOF mass spectrometer. The instrument operated in dual ESI modes (positive and negative ionisation). Chromatographic separation was achieved using an ACQUITY UPLC BEH C18 column (2.1 × 100 mm, 1.7 μm particles), with the column temperature stabilised at 45 °C. The mobile phase consisted of 0.1% formic acid in water (solvent A) and acetonitrile (solvent B). Data were acquired in full-scan mode across a mass-to-charge (m/z) range of 50–1200, with a scan interval of 0.2 s. Detailed gradient elution parameters and mass spectrometry conditions were provided in Supplementary method 3. Raw MS data were processed through MSConvert (v3.0) using centroiding and threshold filtering, following methodologies optimised in our previous work [24, 25].

### Western blot (WB)

Hepatic protein expression profiles were analyzed by WB using standard protocols. Briefly, liver tissue lysates were homogenized in RIPA buffer containing protease/phosphatase inhibitors (Beyotime, China), and protein concentrations were quantified using a bicinchoninic acid (Thermo Scientific). Protein (30 μg/lane) were mixed on 10% SDS-PAGE gels, whereafter removed to PVDF membranes (Millipore). After blocking with 5% BSA, incubated with the following primary antibodies: GPR135 (CST, USA), AMPKα (CST, USA), Phospho-AMPK (CST, USA), ACC1 (Immunoway, China), Phospho-ACC1 (Immunoway, China), and CIDEA (Immunoway, China). GAPDH (Affinity, USA) served as loading control. HRP-labeled Goat Anti-Rabbit lgG (H + L) (Affinity, USA) were applied. Attached membrane with protein was observed through ECL Prime (Tanon, China) and area measured by ImageJ with background subtraction and normalization to GAPDH.

### Reverse transcription-polymerase chain reaction (RT-PCR)

Total RNA was first extracted from intestinal tissues using appropriate kits such as the miRNeasy Kit. Then, the High Capacity cDNA Reverse Transcription Kit was employed to reverse transcribe the RNA into cDNA. Specific primers targeting *GPR41/43, GPR135, AMPK, Cidea, ACC1* were utilized in the RT-PCR process. The PowerUP SYBR Green Polymerase was used to perform real-time PCR amplification. The reaction was carried out according to the manufacturer’s instructions, and the expression levels of *GPR41/43* were determined by analyzing the amplification results. All primers were verified for the production of a single specific PCR product via melting curve analysis to ensure the accuracy of the detection. The relative expression levels of *GPR41/43, GPR135, AMPK, Cidea, ACC1* can be standardized to a reference gene GAPDH to account for variations in sample loading and RNA quality. The PCR primers were provided by Sangon Bioengineering Co., LTD., and all the reagents were provided by CWBIO Biotechnology Co., LTD. The primer sequences were shown in Supplementary Table 1.

### Cell culture and experiment

Cell culture methods were described in Supplementary method 5. To investigate the therapeutic mechanisms of SCFAs (acetic acid: propionic acid: butyric acid = 1: 0.18: 0.09, derived from the proportion structure of fecal SCFAs after QKL administration) in NAFLD, an in vitro steatosis model was established by treating human hepatic stellate cells (LX2) with 660 μM oleic acid (OA) and 330 μM palmitic acid (PA) for 24 h. SCFAs were administered at graded concentrations (100–400 μM) to assess dose-dependent effects. Cell viability was quantified via CCK-8 assay, identifying 100 μM and 200 μM as optimal non-toxic doses for subsequent experiments. Therapeutic efficacy was evaluated through biochemical assays measuring TG and TC levels, complemented by Oil Red O staining to quantify lipid droplet accumulation. Mechanistic insights were derived from RT-PCR analysis of key lipid metabolism regulators (*GPR135*, *AMPK*, *Cidea*, and *ACC1*), with gene expression normalized to *GAPDH*. All procedures adhered to standardized protocols for cell culture, reagent preparation, and molecular analysis to ensure reproducibility.

### Statistical analysis

Results were expressed as mean ± SEM. Statistical analyses and graphs were generated using SPSS 26.0 (Armonk, USA) and GraphPad Prism 8.0.2 (San Diego, USA). Group differences were evaluated using one-way ANOVA followed by Tukey’s post hoc test, with significance set at *P* < 0.05.

## Results

### Chemoprofiling of QKL

**Fig. 1:**
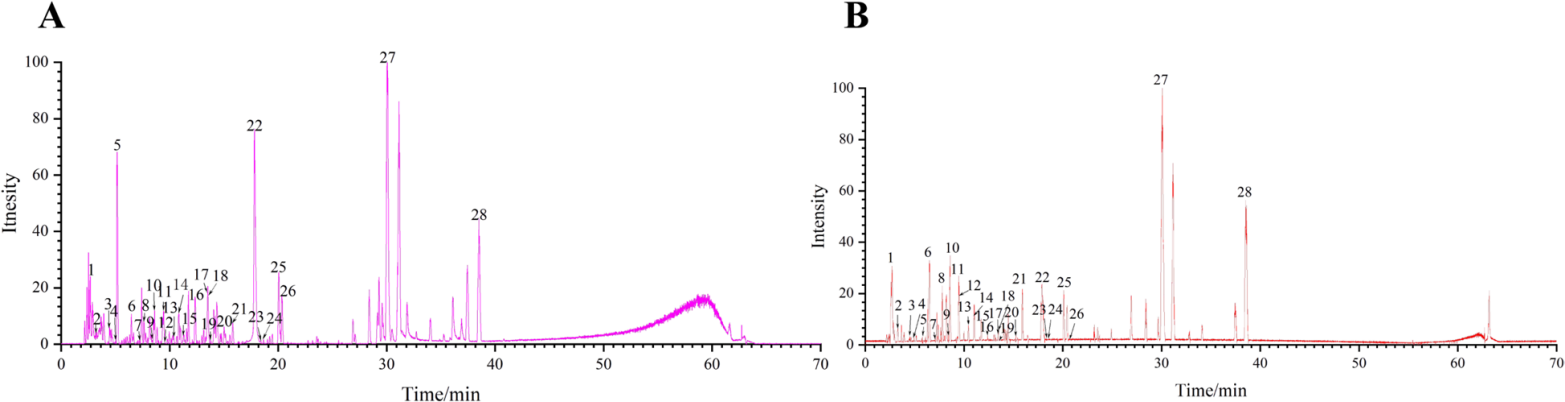
UPLC-TOF/MS chromatogram of Qing-Kai-Ling (QKL) oral liquid: base peak ion chromatograms in (**A**) negative ion mode and (**B**) positive ion mode

Using UPLC-Q-TOF/MS analysis, a total of 28 compounds of the QKL formula were identified, including 4 cholic acids, 4 flavonoids, 6 iridoid terpenoids, 12 organic acids, and 2 other compounds. The positive and negative ion flow chromatography results for the QKL formula were shown in Fig. 1A, B, and the 28 chemical constituents and their molecular formulas and identifications were shown Table 1.

**Table 1:** Compounds identified in QKL by UPLC-TOF/MS

No.	t/min	Formula	Molecular mass (g/mol)	Chemical name	Relative content (%)
1	2.61	C_12_H_22_O_11_	341.11	Fructose	6.58
2	3.19	C_9_H_11_NO_3_	180.07	Tyrosine	1.39
3	4.49	C_16_H_24_O_11_	391.13	Gardenia jasminoides	0.73
4	4.92	C_16_H_22_O_10_	373.11	Geniposide	0.56
5	5.87	C_17_H_24_O_11_	449.30	Hydroxyisogardenia jasminoides	0.69
6	6.33	C_16_H_24_O_10_	375.13	Strychnosidic acid	7.07
7	7.05	C_7_H_6_O_3_	137.04	Salicylic acid	0.51
8	7.64	C_23_H_24_O_15_	549.18	Genipin-1-β-D-gentiobioside	4.93
9	8.42	C_9_H_10_O_5_	197.13	Butyric acid	0.88
10	8.57	C_16_H_18_O_9_	353.09	Chlorogenic acid	6.50
11	9.46	C_24_H_38_O_5_	405.26	3,12-Dihydroxy-7-ketocholanic acid	5.77
12	9.50	C_11_H_12_O_4_	207.07	Caffeoyl ethyl ester	4.23
13	10.43	C_9_H_8_O_4_	179.03	Caffeic acid	1.73
14	10.84	C_25_H_24_O_12_	515.33	Isochlorogenic acid A	2.63
15	11.18	C_17_H_20_O_9_	367.10	Feruloylquinic acid	2.18
16	12.47	C_27_H_30_O_16_	609.16	Rutin	0.85
17	13.44	C_21_H_20_O_12_	463.09	Hypericin	0.97
18	13.48	C_9_H_8_O_3_	163.04	p-Coumaric acid	1.25
19	13.67	C_17_H_26_O_11_	405.23	Monoside	0.50
21	15.92	C_16_H_18_O_9_	353.09	Cryptochlorogenic acid	0.76
20	15.23	C_16_H_18_O_9_	353.09	Neochlorogenic acid	4.65
22	17.97	C_21_H_18_O_11_	445.08	Baicalin	5.03
23	18.45	C_26_H_26_O_12_	529.14	Feruloyl caffeoylquinic acid	0.47
24	18.51	C_10_H_10_O_4_	193.05	Ferulic acid	0.51
25	20.10	C_22_H_20_O_11_	459.09	Scutellarin	4.57
26	20.69	C_24_H_40_O_6_	423.28	3,6,7,12-Tetrahydroxycholanic acid	0.54
27	29.98	C_24_H_40_O_5_	407.28	Cholic acid	21.45
28	38.52	C_24_H_40_O_4_	391.28	Porcine deoxycholic acid	12.09

### QKL treatment alleviated NAFLD symptoms in NAFLD mice

**Fig. 2:**
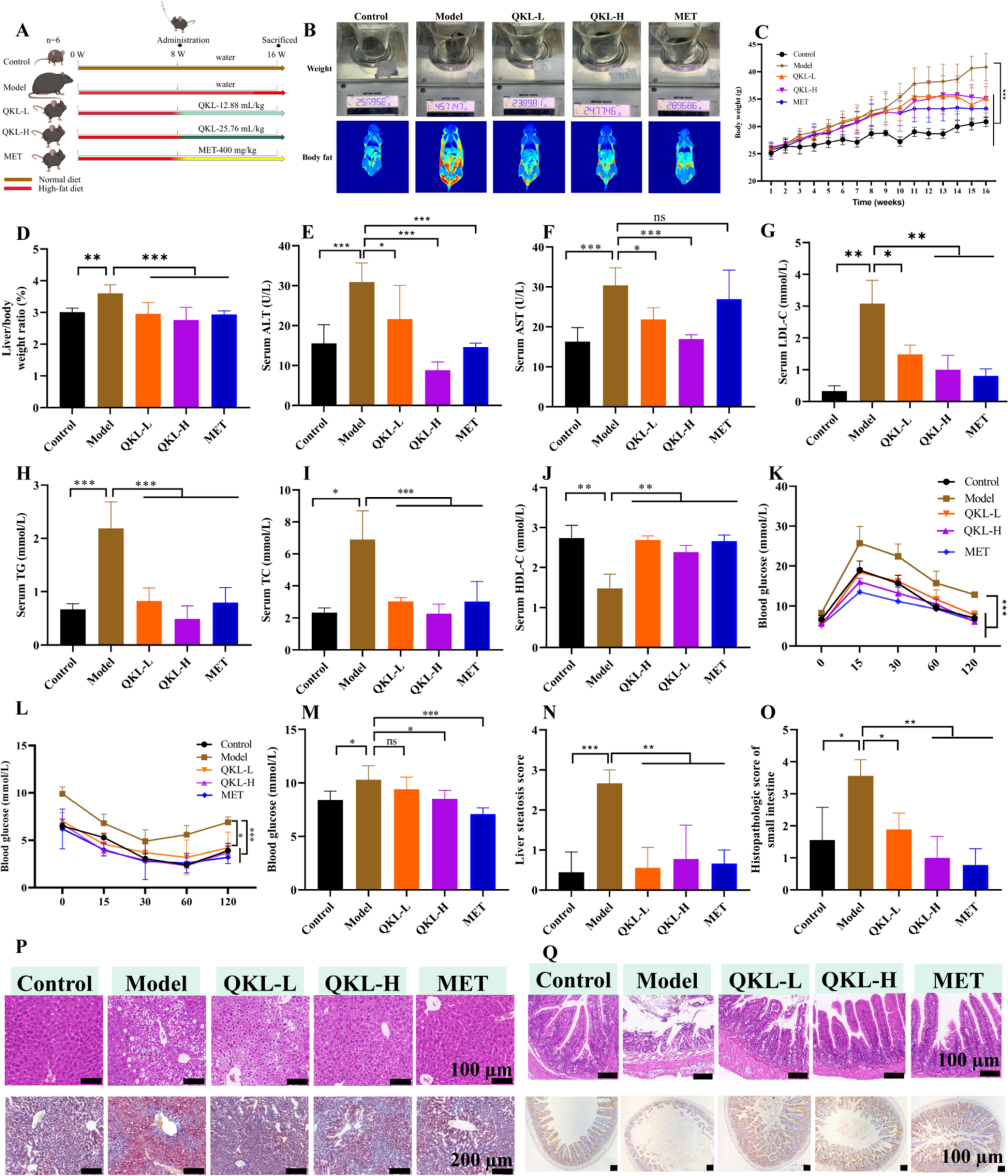
Effect of Qing-Kai-Ling (QKL) oral liquid on ameliorating NAFLD mice. **A** Animal experimental design timeline. **B** Weighing and body fat monitoring. **C** Body weight change. **D** Liver/body weight ratio. The level of **E** ALT, **F** AST, **G** LDL-C, **H** TG, **I** TC, **J** HDL-C in NAFLD mice serum. **K** The oral glucose tolerance test. **L** The insulin tolerance test. **M** Blood glucose. **N** Liver steatosis score. **O** Histopathologic score of small intestine. **P** Liver tissue pathological section of H&E (20 ×) above and oil red below (10 ×). **Q** Small intestine tissue pathological section of H&E above and immunohistochemistry below (20 ×). These data were presented as mean ± SEM (*n* = 6). ^*^P < 0.05*,* ^**^P < 0.01, ^***^P < 0.001 compared with the Model group; one-way ANOVA with a post hoc Tukey test

Initially, to confirm the possible effect impact of QKL on NAFLD, for 16 weeks, we fed mice an HFD, additionally providing QKL/MET supplementation or a control for the last 8 weeks starting from week 9 (Fig. 2A). Consistent with expectations, mice on a HFD increased the liver-to-body weight ratio, total body weight, and fat mass compared to Controls (Fig. 2B–D). Conversely, QKL effectively improved these obesity characteristics, including body weight and surface fat. The AST (*P* < 0.019), ALT (*P* < 0.012), LDL-C (*P* < 0.015), TG (*P* < 0.0001), and TC (*P* < 0.0001) contents in vivo decreased in mice administrated with HFD + QKL-L, QKL-H, and MET (AST, *P* > 0.05) when compared with the Model mice (Fig. 2E–I), whereas HDL-C exhibited an inverse trend (Fig. 2J). The glucose tolerance test (GTT) and the insulin tolerance test (ITT) were also conducted, showing that mice given an HFD treated with QKL had improved insulin sensitivity and glucose tolerance (Fig. 2K–M). Additionally, the liver’s H&E and Oil Red O staining findings showed that the NAFLD mice exhibited steatosis (*P* < 0.005) and an accumulation of lipid droplets (Fig. 2N), and these lesions were significantly (*P* < 0.01) reversed after QKL and MET intervention (Fig. 2P). In addition, the results of H&E and immunohistochemical (MUC-2) of the small intestine demonstrated that the villi, basement and mucosa of the small intestine of NAFLD mice (Fig. 2O, Q). The heat of the mice was detected by thermal imaging, and it was discovered that the model group of mice had a low body temperature, while the Control and administered calories were high (Fig. S1). These results suggested that QKL had an ameliorative effect on metabolic dysfunction, especially steatosis of the liver, in NAFLD mice.

### QKL therapy changed the composition of the NAFLD mice gut microbiota

**Fig. 3:**
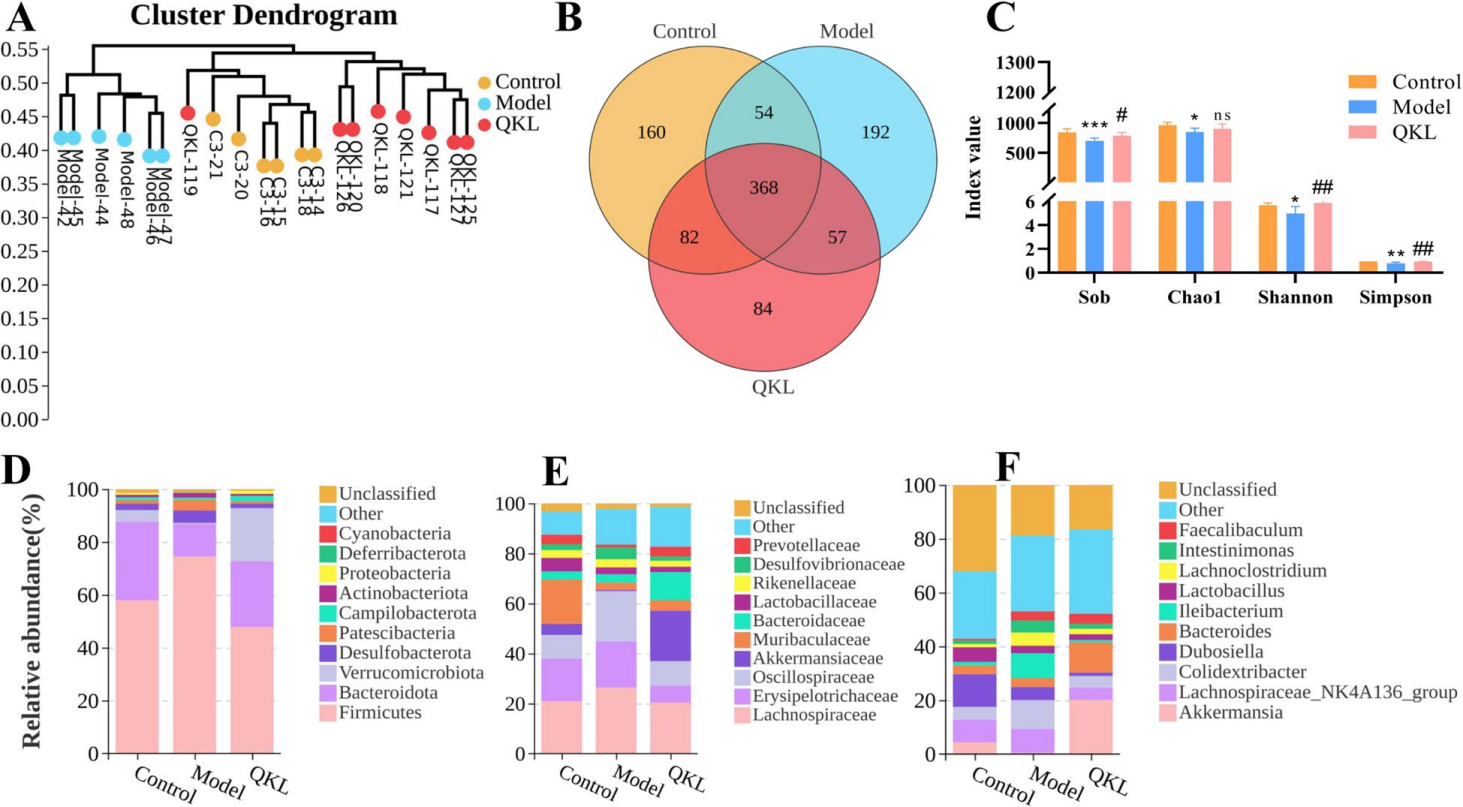
Influence of Qing-Kai-Ling (QKL) oral liquid on gut microbiota in NAFLD mice. **A** Cluster diagram. **B** Venn diagram analysis. **C** Species abundance and diversity index. Bacterial species abundance of **D** Phlyum, **E** Family, **F** Genus. Data were shown as mean ± SEM (*n* = 6), Control versus Model, ^*^P < 0.05*,* ^**^P < 0.01, ^***^P < 0.001. Model versus QKL ^#^*P* < 0.05, ^##^*P* < 0.01, ^###^*P* < 0.001; one-way ANOVA with a post hoc Tukey test

Considering that QKL showed notable anti-NAFLD effectiveness in vivo, its modulation of gut microbiota may underlie therapeutic benefits. Based on pharmacodynamic evaluation, the high-dose QKL group (QKL-H) was selected for subsequent mechanistic exploration. Microbiota construction was analyzed through comparative 16S rRNA sequencing was conducted across the Control, Model, and QKL-H (QKL) groups. 815,337 superior quality reads were produced, resulting in 466 operational taxonomic units (OTUs), which were subsequently annotated. The result of the cluster dendrogram implied that the flora composition of the three groups was different, and the flora structure of the mice in QKL group were closer to the Control (Fig. 3A). We found that 368 common bacteria to all three groups were detected (Fig. 3B). As illustrated in Fig. 3C, the Model group displayed a marked decrease in the microbial communities’ variety and richness in contrast to the Control and QKL groups, evidenced by decreased values in the Sobs and Chao1 indices (richness indices) and Shannon and Simpson indices (diversity indices). Mice with NAFLD exhibited a notable rise in the relative prevalence of Firmicutes and a reduction in Bacteroidota (Phylum). However, this effect at the phylum level was counteracted by QKL treatment (Fig. 3D). In addition, the percentage of Verrucomicrobiota in the Model group significantly decreased in comparison to both the Control and QKL groups. Proportional levels of *Desulfovibrionaceae, Lachnospiraceae*, *Erysipelotrichaceae*, *Oscillospiraceae* were higher in the Model group at the family level when compared to the Control group, while those of *Muribaculaceae*, *Akkermansiaceae* and *Prevotellaceae* were decreased (Fig. 3E). Notably, QKL administration did not reverse the trend of *Desulfovibrionaceae* in NAFLD mice. Compared to the Control, *Lachnospiraceae NK4A136_group*, *Colidextribacter*, *Lleibacterium*, *Lachnoclostridium* and *Intestinimonas* were dramatically improved in the Model group. Conversely, QKL treatment reversed the variations in *Lachnospiraceae NK4A136_group*, *Colidextribacter*, *Lleibacterium*, *Lachnoclostridium* and *Intestinimonas* but higher relative abundances of *Akkermansia* (Genus, Fig. 3F).

**Fig. 4:**
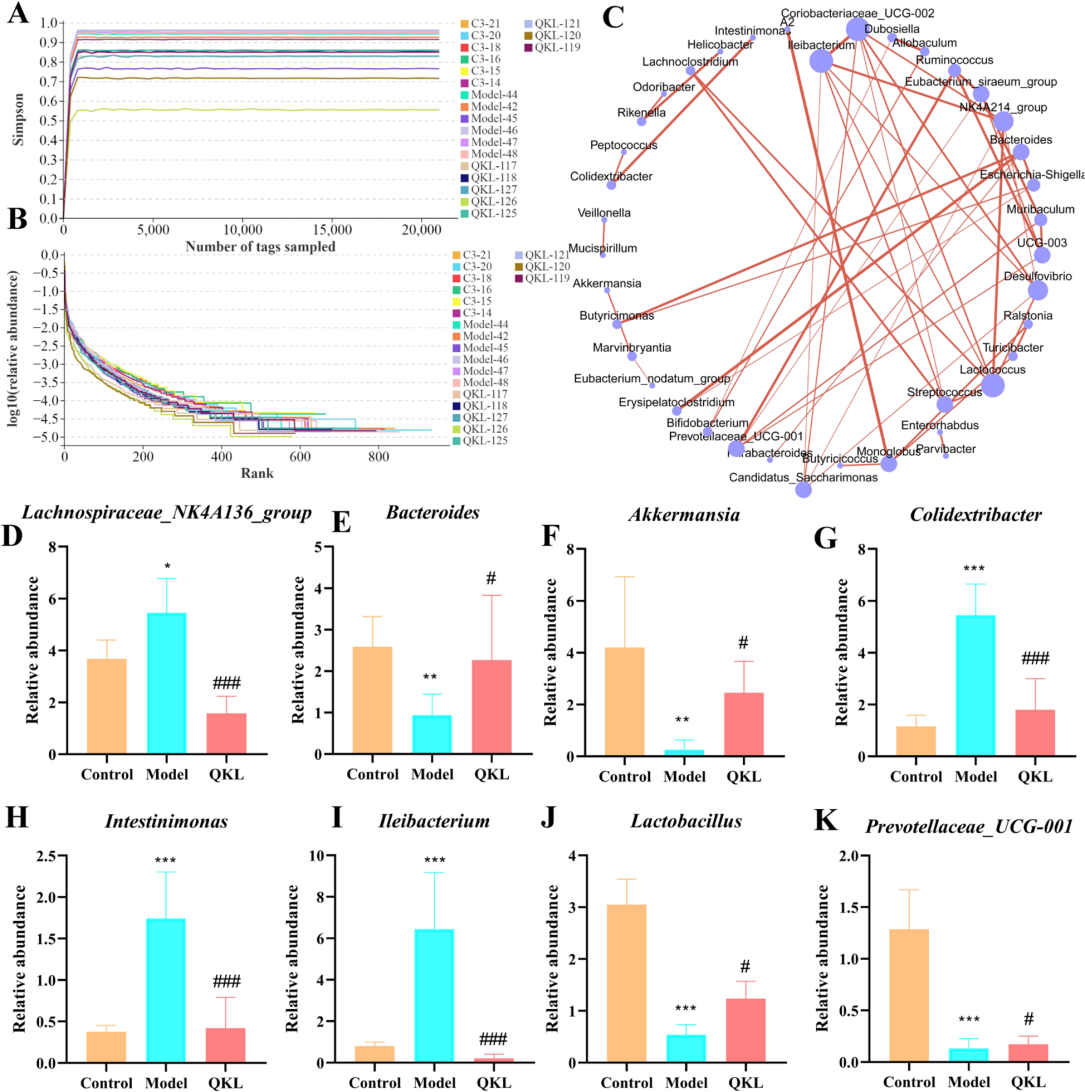
Effect of Qing-Kai-Ling (QKL) oral liquid on gut microbiota composition in NAFLD mice. **A** Rarefaction curve base on Simpson. **B** Rank abundance. **C** Bacterial interaction correlation network at genus. Relative expression abundance of **D** *Lachnospiraceae_NK44136 group*, **E** *Bacteroides,* **F** *Akkermansia*, **G** *Colidextribacter*, **H** *Intestinimonas*, **I** *Ileibacterium*, **J** *Lactobacillus*, **K** *Prevotellaceae_UCG-001* at the generic level. Data were shown as mean ± SEM (*n* = 6), Control versus Model, ^*^P < 0.05, ^**^P < 0.01, ^***^P < 0.001. Model versus QKL ^#^*P* < 0.05*,* ^##^*P* < 0.01, ^###^*P* < 0.001; one-way ANOVA with a post hoc Tukey test

Using the relative proportions of known OTUs in the measured 16S rRNA sequence, a fixed number of reads were randomly selected from each group of samples to get a sparse curve, ensuring the sequencing data quality. As the depth of sequencing increased, the sparse curve rose sharply, suggesting that the community had a significant number of distinct species (Fig. 4A). As the curve began to flatten, it was considered that the depth of sequencing had covered all species, indicating that the sufficient sequencing depth for further study was reasonable to Fig. 4B. To investigate taxonomic associations among species-level differences, a microbial co-occurrence network analysis was performed (Fig. 4C). The analysis identified several keystone taxa, including *Lachnospiraceae_NK4A136_group*, *Bacteroides*, *Akkermansia*, *Colidextribacter*, *Intestinimonas*, *Ileibacterium*, and *Prevotellaceae_UCG-001*, as hub species with highest network centrality (Degree > 10, *P* < 0.05). Subsequently, we quantified the relative abundance of these putative keystone taxa. QKL treatment significantly enriched potentially beneficial bacteria, including *Bacteroides* (*P* = 0.047), *Akkermansia* (*P* = 0.048), and *Prevotellaceae_UCG-001*, in the gut microbiota of NAFLD mice (Fig. 4E–F, J, K, P < 0.05), while suppressing putative pathobionts such as *Lachnospiraceae_NK4A136_group*, *Colidextribacter*, *Intestinimonas*, and *Ileibacterium* (Fig. 4D, G–I, P < 0.0001). Collectively, QKL intervention significantly restructured the makeup of gut microbiota in mice with NAFLD, indicating that QKL may alleviate NAFLD progression through gut microbiota modulation.

### QKL fecal microbiota transplants revealed the effect of QKL in NAFLD mice involving microbiota-dependent

**Fig. 5:**
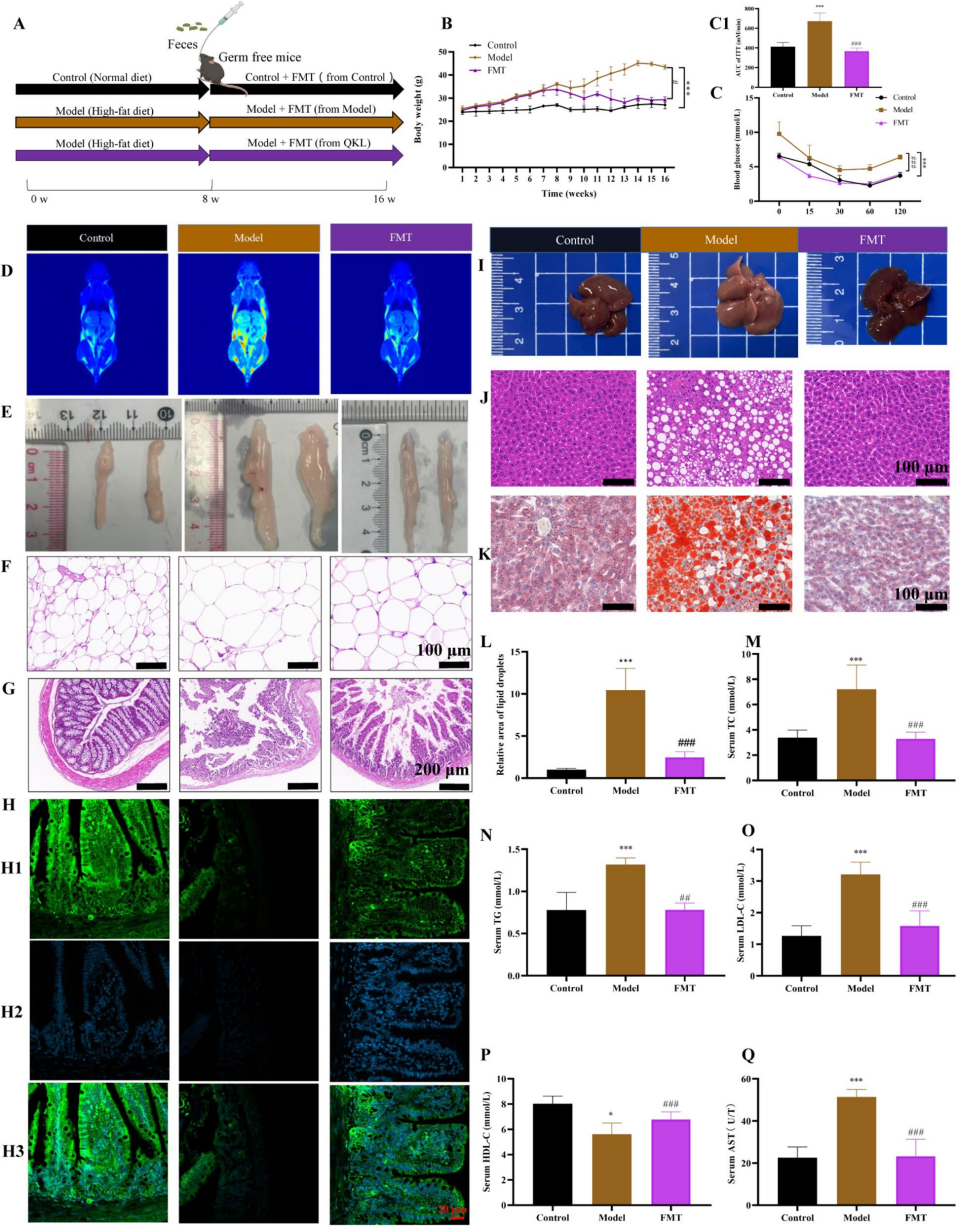
Effects of fecal transplantation (FMT) after treatment with Qing-Kai-Ling (QKL) oral liquid on mice with NAFLD. **A** FMT animal experiment design flow chart. **B** Body weight change. **C** Insulin resistance. **D** Fat distribution monitoring. **E** White fat. **F** H&E of white fat. **G** H&E of small intestine (5 ×). **H** Immunofluorescent image of occludin protein in the small intestine (40 ×), H1: occludin, H2: dapi, H3: merge. **I** Liver. **J** H&E of liver (10 ×). **K** Liver oil red (10 ×). **L** Relative area of lipid droplets. **M** Serum TC. **N** Serum TG. **O** Serum LDL-C. **P** Serum HDL-C. **Q** Serum AST. Data were shown as mean ± SEM (*n* = 6), Control versus Model, ^#^*P* < 0.05*,* ^##^*P* < 0.01, ^###^*P* < 0.001. Model versus FMT, ^*^P < 0.05*,* ^**^P < 0.01, ^***^P < 0.001; one-way ANOVA with a post hoc Tukey test

Although QKL exhibited microbiota-modulating properties associated with therapeutic benefits, we further investigated whether the gut microbiota mediated its anti-NAFLD effects. To validate this hypothesis, an antibiotic cocktail-induced gut microbiota depletion in the NAFLD mice was constructed before administering the intervention. Successful depletion of gut bacteria was confirmed by horizontal agarose gel electrophoresis (Fig. S2). To validate the microbiota-dependent mechanism of QKL, we performed FMT by transferring fecal suspensions from QKL-treated donor mice into NAFLD ones. and then analyzed the indicators associated with colitis (Fig. 5A). Following FMT from QKL-treated donors, recipient mice with NAFLD reduced body weight at the study endpoint compared to the Model group (Fig. 5B). AUC of insulin tolerance test (ITT), were elevated in NAFLD mice while significantly decreased after FMT (Fig. 5C). Subsequently, we assessed the adipose tissue remodeling in NAFLD mice following FMT. The results of the magnetic resonance adipography and histopathological analysis revealed that FMT intervention significantly attenuated hepatic steatosis and visceral adiposity (Fig. 5D, E). Quantitative histomorphometric analysis of white adipose tissue (WAT) via H&E staining demonstrated a marked reduction in adipocyte diameter (Fig. 5F), suggesting that microbiota-driven suppression of adipocyte hypertrophy contributed to the anti-NAFLD effects of QKL. Small intestinal sections via H&E staining revealed that FMT preserved ileal villus integrity in NAFLD mice, with significant increased in villi integrity and small intestine recess depth compared to the Model group (Fig. 5G). Immunofluorescence quantification demonstrated elevated expression of tight junction protein occludin (Fig. 5H), suggesting that microbiota-mediated restoration of gut barrier integrity contributes to the attenuation of NAFLD progression. Critically, gross morphological evaluation revealed that FMT attenuated hepatomegaly in NAFLD mice (Fig. 5I). Histopathological analysis further demonstrated that FMT intervention significantly reduced hepatic steatosis, as evidenced by decreased lipid droplet deposition (Fig. 5J). Oil Red O indicating microbiota-driven mitigation of hepatic lipid accumulation (Fig. 5K). Moreover, the contents of TG, TC, LDL-C, and AST were elevated in the Model group but significantly decreased following FMT administration (Fig. 5M-O, Q). However, HDL-C level showed an increase after treatment (Fig. 5P). The above results were in agreement with previous QKL treatment results. It was revealed that the effect of QKL on NAFLD was dependent on the regulation of the gut microbiota.

### QKL regulated SCFAs levels in NAFLD mice

**Fig. 6:**
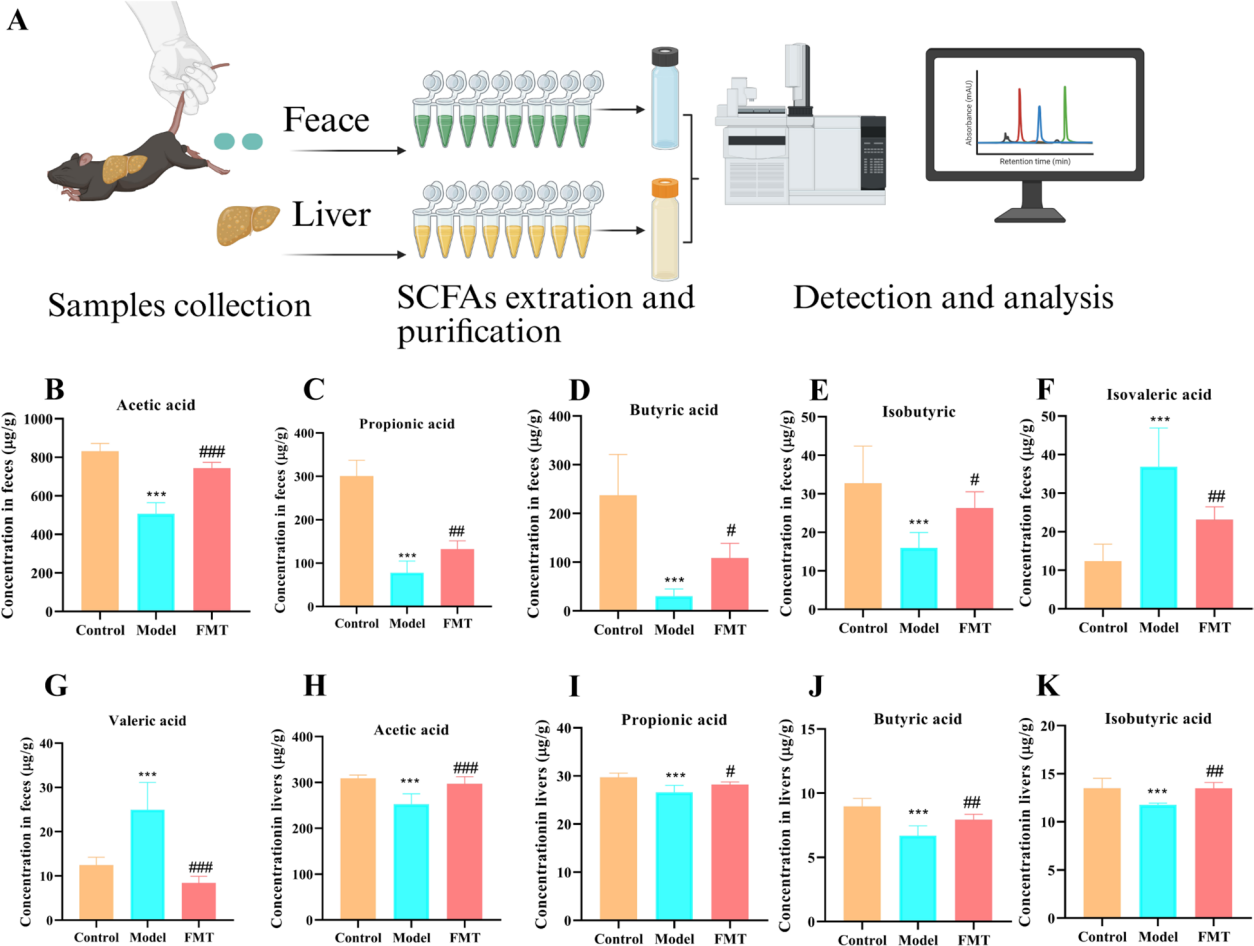
Short-chain fat acids (SCFAs) levels in feces and liver of NAFLD mice treated with Qing-Kai-Ling (QKL) oral liquid. **A** Flowchart depicting the process of extracting, purifying, detecting, and analyzing SCFAs. Concentration of **B** acetic acid, **C** propionic acid, **D** butyric acid, **E** isobutyric acid, **F** isovaleric acid, **G** valeric acid in feces. Concentration of **H** acetic acid, **I** propionic acid, **J** butyric acid, **K** isobutyric acid in livers. Data were shown as mean ± SEM (*n* = 6), Control versus Model, ^*^P < 0.05*,* ^**^P < 0.01, ^***^P < 0.001. Model versus FMT ^#^*P* < 0.05*,* ^##^*P* < 0.01, ^###^*P* < 0.001; one-way ANOVA with a post hoc Tukey test

Gut microbiota dysulation in NAFLD mice was accompanied by systemic depletion of SCFAs, as revealed by gas chromatography quantification of six fecal metabolites (acetic acid, propionic acid, butyrate, isobutyrate, valerate, isovalerate) and four hepatic SCFAs (Fig. 6A). In Fig. 6B–G, the result shown that FMT from QKL-treated donors significantly upregulated acetic acid (*P* < 0.0001), propionic acid (*P* = 0.0091), butyrate (*P* = 0.036) and isobutyrate (*P* = 0.0027), while downregulating valerate (*P* < 0.0001) and isovalerate acid (*P* = 0.005). Moreover, hepatic profiling demonstrated parallel increased in acetic acid, propionic acid, and butyrate isoforms post-FMT (*P* < 0.05), though valeric acid isoforms remained undetectable in liver tissue (Fig. 6H–K). Overall, this results suggested microbiota-driven SCFAs remodeling contributed to QKL’s therapeutic effects through enterohepatic metabolite circulation.

### FMT attenuated NAFLD via AMPK signaling pathway activation and SCFA receptor regulation

**Fig. 7:**
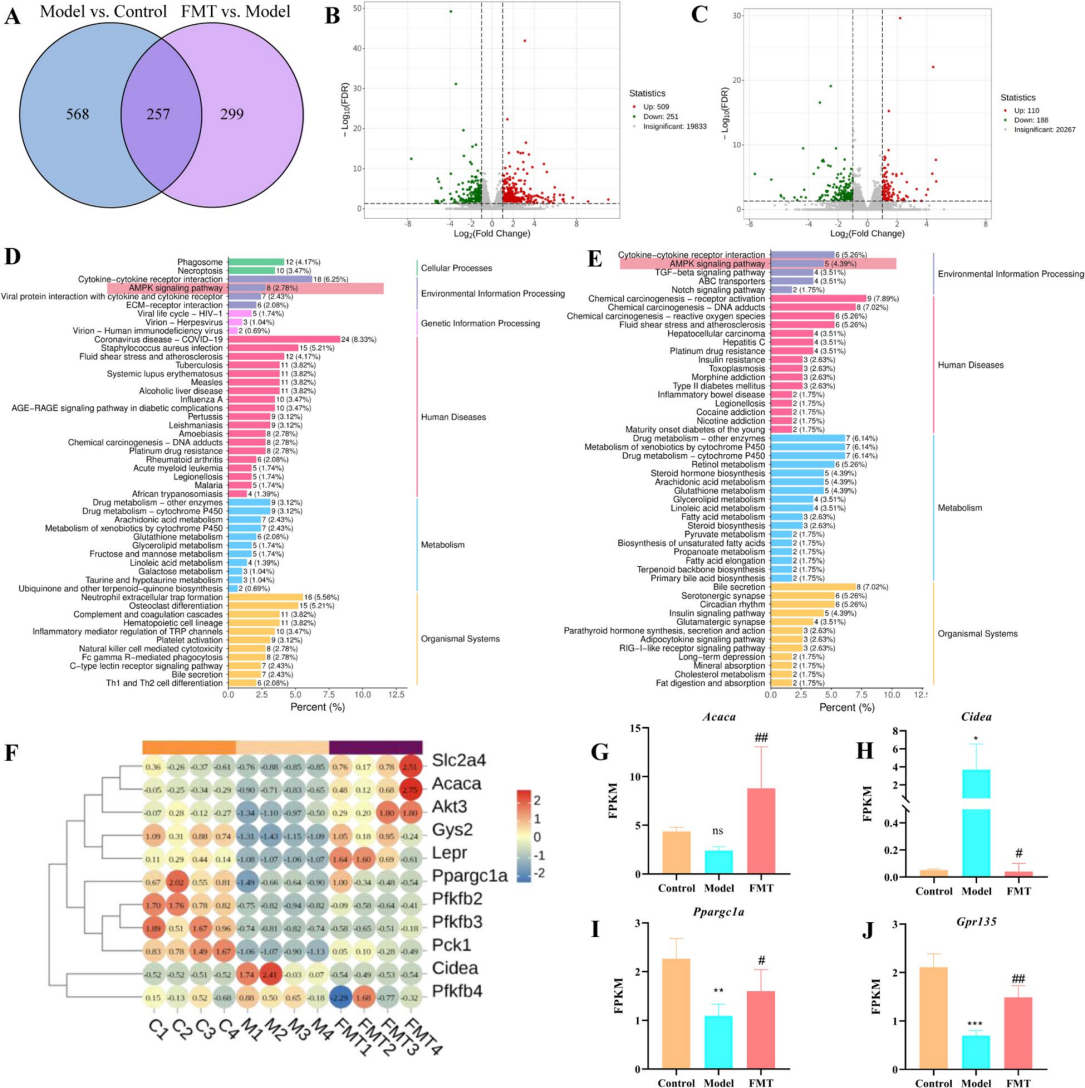
Transcriptomics revealed potential targets and pathways for fecal transplantation (FMT) after treatment with Qing-Kai-Ling (QKL) oral liquid in the liver of mice with NAFLD. **A** Venn diagram of common differentially expressed genes among the three groups. Volcano plot of inter-group differentially expressed genes between **B** Control versus Model, **C** Model versus FMT. KEGG of **D** Control versus Model, **E** Model versus FMT. **F** Heatmap of key target genes enriched in the AMPK signaling pathway. Relative expression levels of key differentially expressed genes of **G** *Acaca*, **H** *CIDEA*, **I** *Ppargc1a*, **J** *Gpr135.* Data were shown as mean ± SEM (*n* = 4), Control versus Model, ^*^P < 0.05*,* ^**^P < 0.01, ^***^P < 0.001. Model versus FMT ^#^*P* < 0.05, ^##^*P* < 0.01, ^###^*P* < 0.001; one-way ANOVA with a post hoc Tukey test

**Fig. 8:**
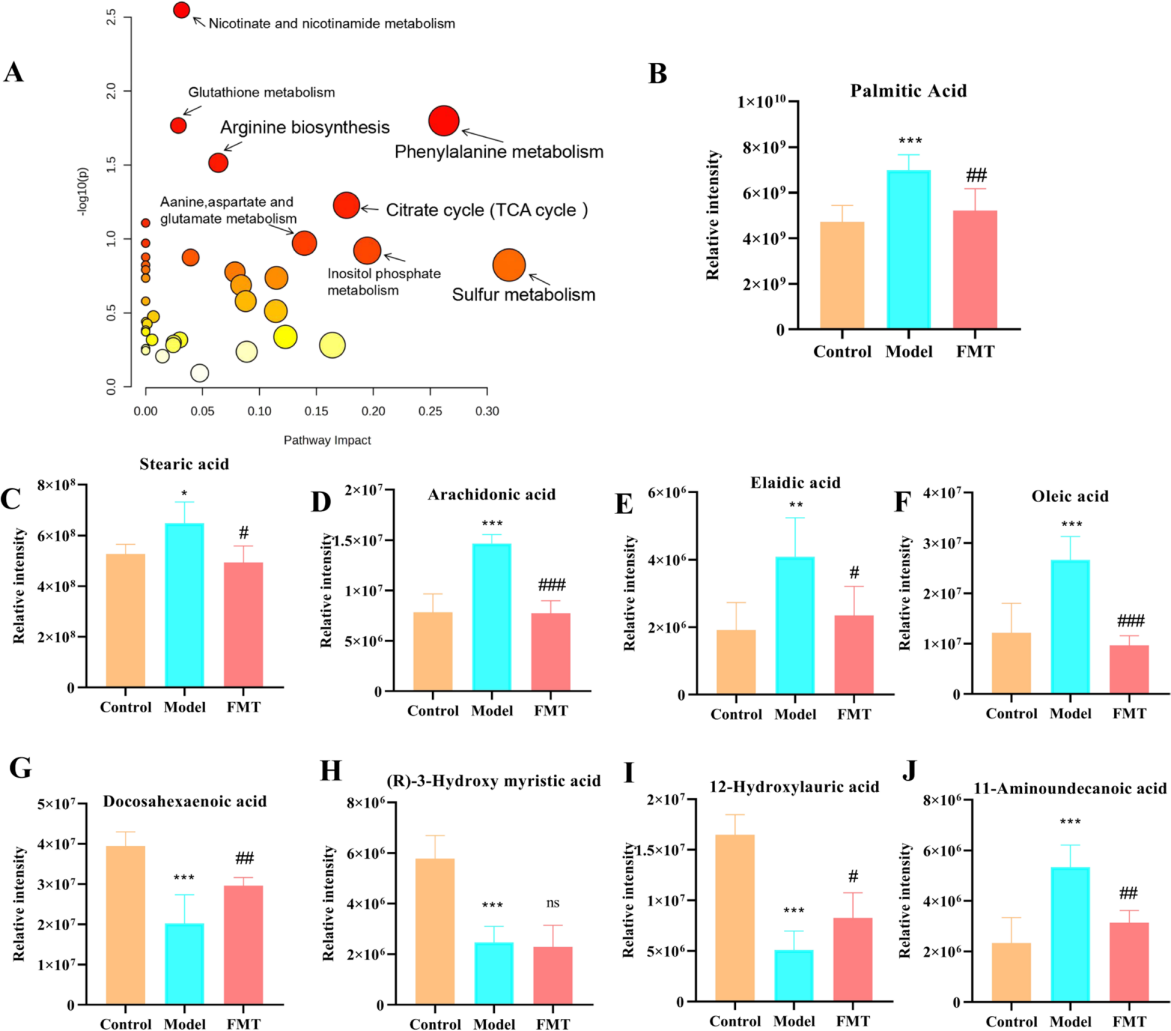
Effects of Qing-Kai-Ling (QKL) oral liquid on representative fatty acids metabolites of livers in NAFLD mice. **A** A major metabolit bubble enrichment diagram of KEGG. The relative intensity of **B** Palmitic acid, **C** Stearic acid, **D** Arachidonie acid, **E** Elaidic acid, **F** Oleic acid, **G** Docosahexaenoic acid, **H** (R)-3-Hydroxy myristic acid, **I** 12-Hydroxylauric acid, **J** 11-Aminoundecanoic acid. Data were shown as mean ± SEM (*n* = 6), Control versus Model, ^*^P < 0.05, ^**^P < 0.01, ^***^P < 0.001. Model versus FMT ^#^*P* < 0.05, ^##^*P* < 0.01, ^###^*P* < 0.001; one-way ANOVA with a post hoc Tukey test

Transcriptome analysis of hepatic tissues was conducted to elucidate the pathways by which QKL mitigates NAFLD. Venn analysis identified 257 overlapping genes across all three groups (Fig. 7A). Volcano plots revealed 541 upregulated and 284 downregulated genes in the Control versus Model groups (Fig. 7B), while 267 upregulated and 289 downregulated genes were observed in the FMT versus Model comparison (Fig. 7C). Analysis of differentially expressed genes using the KEGG pathway highlighted key metabolic pathways associated with NAFLD progression, including the AMPK signaling pathway, linoleic acid, glycerolipid, arachidonic acid, and cholesterol metabolism (Fig. 7D, E). In Fig. 7F, correlation heatmap analysis prioritized AMPK-related targets involved in fatty acid metabolism (e.g., *Acaca*, *Ppargc1a*, *CIDEA*). Post-FMT intervention significantly upregulated *Acaca* (*P* = 0.0098) and *Ppargc1a* expression (*P* = 0.0167) while downregulating *CIDEA* (*P* = 0.021) (Fig. 7G-I). Furthermore, hepatic *GPR135* (*P* = 0.0012) expression, a receptor for SCFAs, was reduced in the Model group compared to Control and partially restored by FMT (Fig. 7J). Collectively, these findings suggested FMT ameliorated NAFLD by modulating AMPK-driven fatty acid metabolism and reactivating SCFA receptor signaling (Fig. 8).

### Effects of FMT on SCFAs receptors and key protein expressions in NAFLD mice

**Fig. 9:**
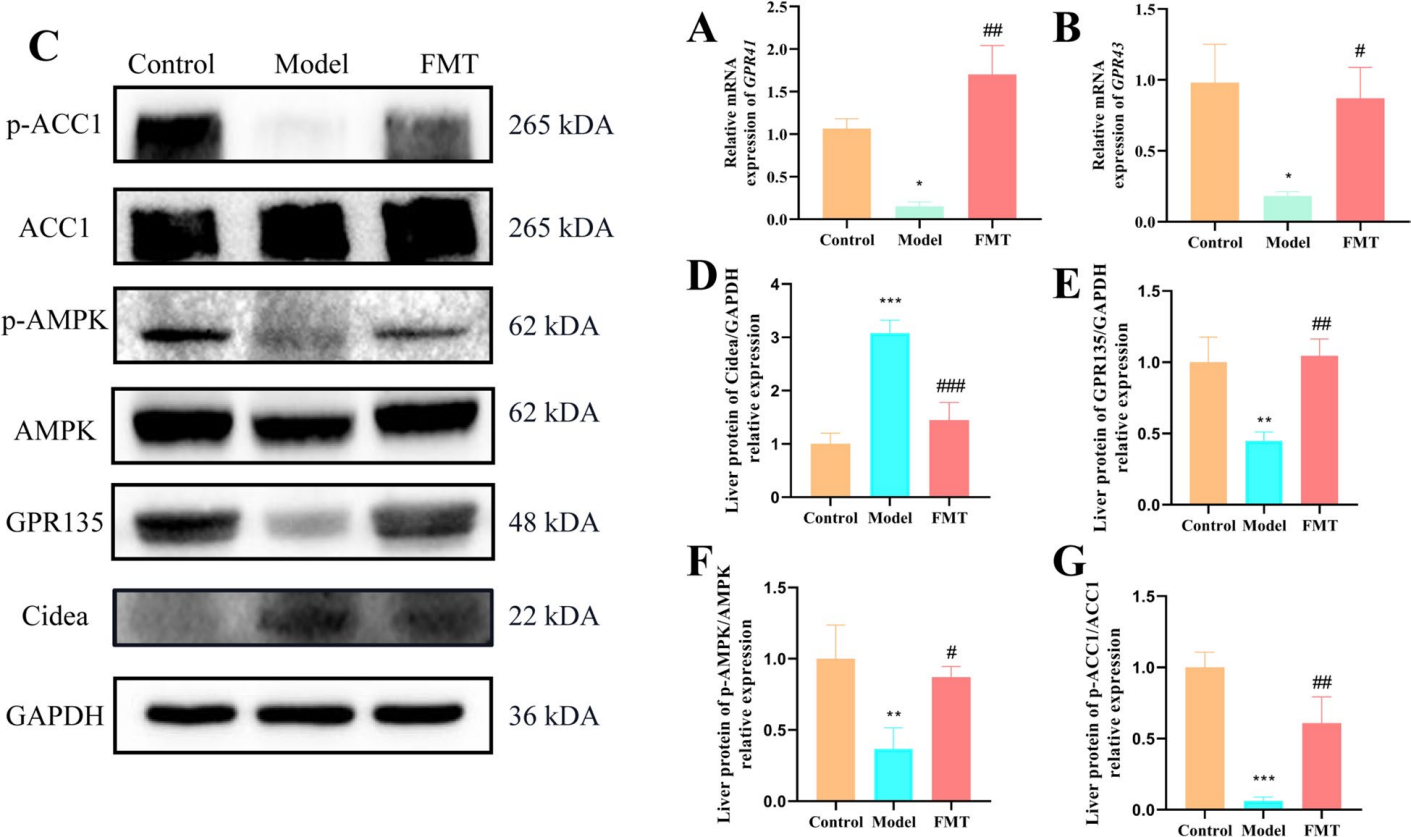
Qing-Kai-Ling (QKL) oral liquid through gut microbes regulated the GPR135/AMPK/ACC1 signaling pathway. Relative mRNA expression of *GPR41* **A** and *GPR43* **B** in small intestine. **C** Protein immunoblotting. Liver proteins relative expression of the **D** CIDEA, **E** GPR135, **F** p-AMPK/AMPK, **G** p-ACC1/ACC1. Data were shown as mean ± SEM (*n* = 3), Control versus Model, ^*^P < 0.05*,* ^**^P < 0.01, ^***^P < 0.001. Model versus FMT ^#^*P* < 0.05*,* ^##^*P* < 0.01, ^###^*P* < 0.001; one-way ANOVA with a post hoc Tukey test

Using RT—PCR and WB, the gene expression of SCFAs receptors *GPR41/43* and the protein expression of CIDEA, GPR135, p-AMPK/AMPK, and p-ACC1/ACC1 was assessed for each group, with the findings illustrated in Fig. 9. The RT-PCR results showed that the relative gene expression level of the short-chain fatty acid receptor *GPR41* (*P* = 0.002) and *GPR43* (*P* = 0.041) in the small intestine of NAFLD mice was significantly lower than that of the normal group, and was upregulated after FMT intervention (Fig. 9A, B). The WB statistical result indicated that following the FMT intervention could down-regulation of CIDEA protein expression in the NAFLD mice liver (Fig. 9C, P = 0.0005). Conversely, the levels of GPR135 (*P* = 0.0022), p-AMPK/AMPK (*P* = 0.0183), and p-ACC1/ACC1 (*P* = 0.003) proteins were found to be significantly up-regulated (Fig. 9D–G). These findings implied that FMT influenced specific protein expressions associated with hepatic steatosis development and lipid metabolism.

### Impact of FMT on hepatic fatty acid metabolism in NAFLD mice

To continue observing alterations in hepatic fatty acid metabolism in mice with NAFLD after the FMT intervention, untargeted metabolomics was employed to analyze alterations in hepatic fatty acid levels. The results showed that KEGG-enriched metabolic pathways primarily included phenylalanine, citrate cycle (TCA cycle), sulfur, inositol phosphate, arginine biosynthesis, glutathione, nicotinate and nicotinamide, and alanine, aspartate, and glutamate metabolism (Fig. 8A). Nine fatty acids exhibited altered response values post-FMT intervention. Compared to the model group, six fatty acids (palmitic acid, stearic acid, arachidonic acid, elaidic acid, oleic acid, 12-hydroxylauric acid, and 11-aminoundecanoic acid) were significantly downregulated after FMT treatment (*P* < 0.05) (Fig. 8B–F, I, J). In contrast, docosahexaenoic acid (DHA) showed an opposite trend (Fig. 8G). Additionally, in comparison to the control group, the DHA response value in the liver of the model group was dramatically downregulated, but no significant upregulation was observed after FMT intervention (*P* > 0.05) (Fig. 8H). These results indicated that FMT intervention modulated fatty acid metabolism in NAFLD mice, with key pro-lipogenic fatty acids (e.g., palmitic acid) being suppressed, while DHA dynamics may reflect incomplete restoration of anti-inflammatory pathways, warranting further investigation into the gut-liver axis mechanisms.

### Effects of SCFAs on NAFLD via AMPK-Cidea/ACC1 axis in LX2 model

**Fig. 10:**
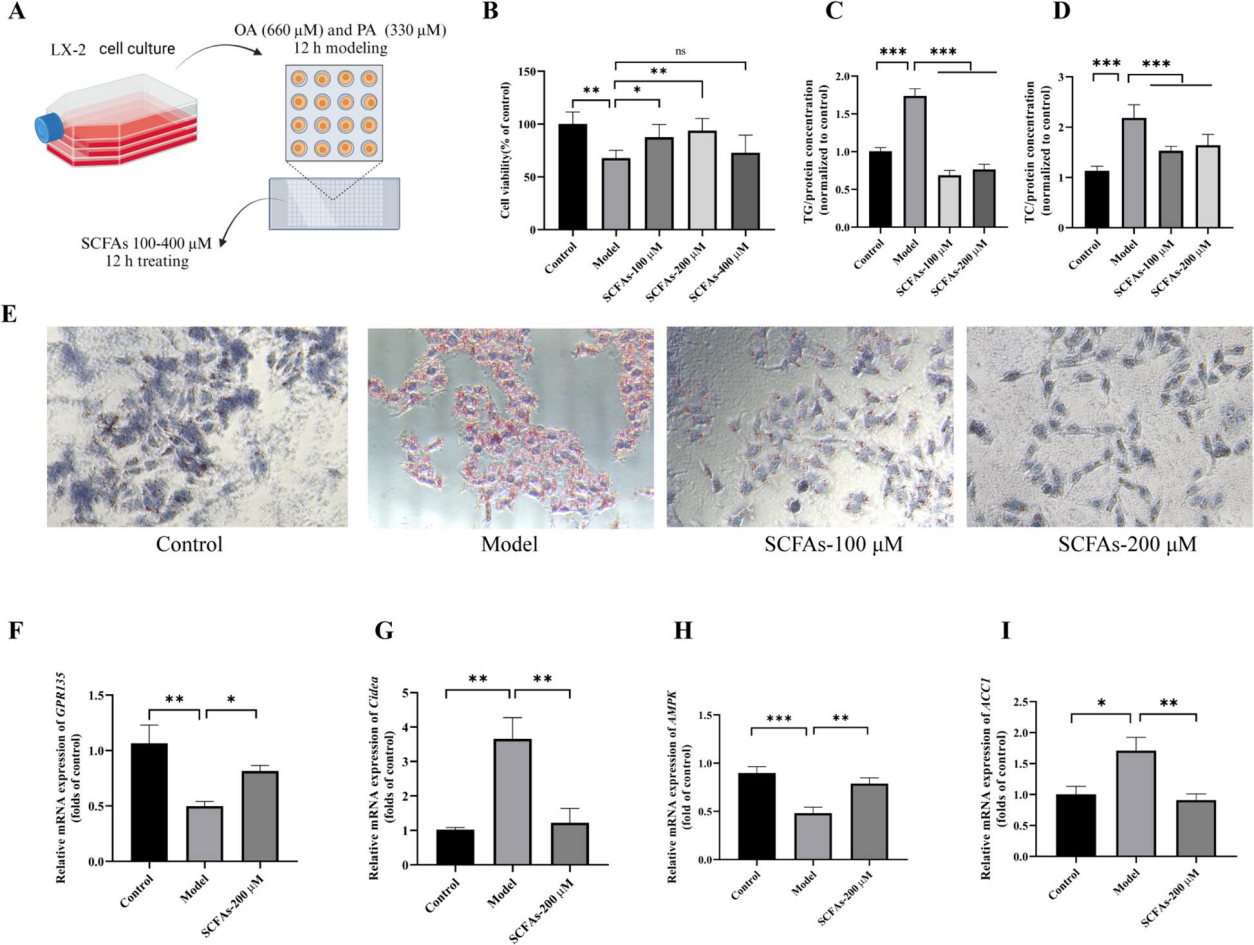
Effects of short-chain fat acids (SCFAs) levels in feces of NAFLD mice treated with Qing-Kai-Ling (QKL) oral liquid in vitro*.* **A** Cell experiment process. **B** Cell viability. Concentration of TG **C** and TC **D**. **E** Cell Oil Red O. Relative mRNA expression of **F** *GPR135*, **G** *Cidea*, **H** *AMPK*, **I** *ACC1*. Data were shown as mean ± SEM (*n* = 6), compared with the Model group, ^*^P < 0.05, ^**^P < 0.01, ^***^P < 0.001; one-way ANOVA with a post hoc Tukey test

To investigate the therapeutic potential of SCFAs and explore their molecular targets in NAFLD, we established an in vitro NAFLD model using LX2 cells treated with 660 μM oleic acid (OA) combined with 330 μM palmitic acid (PA). Subsequent SCFA administration was performed to evaluate its therapeutic efficacy, as outlined in the experimental workflow (Fig. 10A). Initial CCK-8 assays revealed that SCFA treatment at concentrations of 100 μM and 200 μM significantly improved cell viability in the NAFLD model (Fig. 10B). These two optimal doses were therefore selected for subsequent pharmacological evaluations. Notably, our therapeutic assessment demonstrated that SCFAs effectively reduced TG and TC accumulation, with a parallel decrease in lipid droplet formation (Fig. 10C–E, P < 0.0001). The observed therapeutic effects exhibited a distinct dose-dependent pattern, suggesting concentration-responsive biological activity.

Mechanistic exploration through RT-PCR analysis revealed that SCFA treatment upregulated mRNA expression levels of *GPR135* (*P* = 0.045) and *AMPK* (*P* = 0.0082) in a concentration-dependent manner (Fig. 10F, H). Conversely, we observed significant downregulation of lipid metabolism-related genes, including *Cidea* (*P* = 0.0021) and *ACC1*(*P* = 0.0027) (Fig. 10G, I). These transcriptional modifications collectively indicate that SCFAs may ameliorate hepatic steatosis through dual regulatory mechanisms: enhancing energy metabolism via AMPK activation while concurrently inhibiting lipogenic pathways.

## Discussion

NAFLD represented a significant phase in the advancement of chronic liver diseases, potentially resulting in non-alcoholic steatohepatitis, liver fibrosis, cirrhosis, and ultimately hepatocellular carcinoma [1]. Although numerous pathologic variables contribute to the condition, effective therapy choices for NAFLD remain constrained. Therefore, the creation of innovative treatment drugs for NAFLD possesses considerable clinical importance. QKL oral liquid, a traditional Chinese medicine formulation, contained multiple herbal ingredients with notable anti-inflammatory, antioxidant, and hepatoprotective properties [19]. The active ingredients in QKL oral liquid included baicalin, cholic acid, porcine deoxycholic acid, and chlorogenic acid [26]. Previous studies had shown that baicalin improved immune function by upregulating SCFA-producing bacteria such as *Prevotellaceae* [27]. Meanwhile, research demonstrated that upregulation of SCFAs, cholic acid, and deoxycholic acid levels enhanced antioxidant capacity and reduced fat deposition [29–31]. Moreover, chlorogenic acid was found to improve intestinal barrier integrity and reduce hepatic fat accumulation by increasing SCFA levels, particularly butyric acid [32].

Notably, earlier studies had demonstrated the therapeutic effects of QKL in conditions such as acute lung injury and inflammation-related diseases. Recent investigations further suggested its potential in preventing and treating NAFLD through modulation of liver and gut health [18, 33]. However, a critical gap remains: prior studies on QKL and NAFLD only provided correlational evidence (e.g., gut-liver association) and did not experimentally validate whether and how QKL alleviates NAFLD via gut microbiota remodeling, a key mechanism increasingly recognized as central to NAFLD pathogenesis [2–5]. Clinically, the progression of NAFLD, particularly in individuals with fatty liver, was closely linked to AMPK-ACC1 activity [34, 35]. However, whether QKL alleviated NAFLD by regulating the gut microbiota had not been thoroughly investigated. Our work addresses these gaps: we provide novel causal evidence that QKL mitigates NAFLD by dual mechanisms—modulation of hepatic oxidative stress/AMPK pathway, and remodeling of gut microbiota. HFD could cause mice to overnutrition, induce excessive fat synthesis in the body, and damage the animals’ livers, leading to NAFLD [36]. However, treatment with QKL and MET in NAFLD mice caused a more pronounced reduction in body and liver weight and peripheral fat. Previous studies have shown that NAFLD not only damaged the liver but also destroyed the intestinal barrier function of the small intestine, increasing the accumulation of the fat-produced cells [37, 38]. Actually, we investigated that QKL could prevent liver and small intestine impairment in NAFLD mice. The observed decrease in liver weight may be attributed to an increase in liver lipid metabolism, including TG, TC, and LDL-C [39], this was consistent with our results.

The liver lower-lipid effects of QKL could possibly be related to the regulation of gut microenvironmental disturbances. The gut microbiota could modulate gut permeability and SCFA production, both of which influenced liver health [40]. A healthy gut microbiota diminishes liver exposure to inflammatory cytokines and harmful metabolites (e.g., lipopolysaccharides, LPS), which were strongly associated with NAFLD development and progression [41]. The gut microbiota was predominated by Firmicutes and Bacteroidota at the phylum level, and their ratio was critical in NAFLD pathogenesis. A reduced Firmicutes/Bacteroidota ratio was often linked to a lower risk of NAFLD [42]. Key SCFA-producing bacteria (*Bacteroides, Lactobacillus,* and *Akkermansia*) was known to benefit gut and liver health [43, 44]. Most studies only observed correlations between microbiota changes and NAFLD improvement (e.g., increased *Akkermansia*) but lacked experimental validation of whether microbiota remodeling causes NAFLD alleviation [42, 44]; Meanwhile, no study has specifically investigated how QKL targets these microbiota taxa or their metabolites (e.g., SCFAs) in NAFLD. The QKL treatment altered the gut microbiota in NAFLD mice, increasing short-chain fatty acid levels and restoring the mucosal barrier.

Compared to previous reports that HFD enriches Firmicutes and depletes SCFA-producing taxa [47], we show that QKL treatment specifically reduces the Firmicutes/Bacteroidota ratio (restoring it to near-normal levels) and upregulates the abundance of key beneficial taxa (*Akkermansia*, *Bacteroides*, *Muribaculaceae*) in NAFLD mice. This is distinct from non-specific microbiota modulators (e.g., probiotics) that only increase 1–2 beneficial taxa [43], as QKL targets a panel of taxa linked to NAFLD pathogenesis. For the first time, we use FMT to confirm that QKL’s anti-NAFLD effects are microbiota-dependent, a step rarely included in previous QKL-related studies [18, 33] or even some NAFLD microbiota studies [42]. This validates that microbiota remodeling is not just a byproduct of NAFLD improvement, but a driver. We further connect QKL-enriched taxa to their functional effects: *Akkermansia* (and its metabolite propionate) is known to protect the intestinal barrier in ulcerative colitis [45], and we extend this to show it mitigates NAFLD-induced gut damage; *Bacteroides* and *Muribaculaceae* are associated with reduced hepatic fat accumulation [46], which aligns with our finding that QKL increases fecal/hepatic SCFA levels (acetic acid, propionate, butyrate)—directly linking microbiota changes to metabolic benefits. Additionally, we identify QKL’s suppression of putative pathobionts (*Lleibacterium*, *Colidextribacter*, *Intestinimonas, Lachnospiraceae_NK4A136_group*). While *Colidextribacter* and *Lachnospiraceae_NK4A136_group* are known to be enriched in HFD-fed mice [47], and *Bifidobacterium* supplementation suppresses *Intestinimonas* to alleviate hepatic steatosis [48], *Lleibacterium* has only been proposed as a negative marker of mucosal barrier integrity [49]. Our study is the first to associate its suppression with NAFLD improvement, providing a new potential target for NAFLD therapeutics.

Collectively, QKL alleviated NAFLD by restoring gut microbiota balance via enriching SCFA-producing taxa (e.g., *Akkermansia, Bacteroides*) and suppressing pathobionts (e.g., *Colidextribacter*). This microbiota remodeling likely enhanced intestinal integrity, reduced pathogen translocation to the liver, and inhibited hepatic lipid accumulation.

FMT alleviated NAFLD via AMPK signalling and SCFA receptor regulation. Our transcriptome analysis of hepatic tissues revealed that FMT attenuated NAFLD through the activation of the AMPK signalling pathway and regulation of SCFA receptors. Similar to previous studies, activation of the AMPK pathway has been reported to play a crucial role in regulating lipid metabolism [50]. As previous reports demonstrated that AMPK activation can suppress lipogenesis and promote fatty acid oxidation [50, 51], which was consistent with our findings that FMT upregulated *Acaca* and *Ppargc1a*, genes involved in fatty acid metabolism, and downregulated *CIDEA*. Moreover, the restoration of the SCFA potential receptor *GPR135* expression in the liver after FMT intervention was also significant. However, a key innovation of our study is linking FMT to SCFA receptor regulation: we found that FMT restores the expression of GPR135 (a potential SCFA receptor) in the liver, which is downregulated in NAFLD mice. While SCFAs are known to regulate energy homeostasis and lipid metabolism via their receptors [52, 53], prior FMT study in NAFLD focused only on SCFA levels (not receptors) [44] and our work bridges this gap by showing that FMT improves NAFLD not just by increasing SCFAs, but by reactivating SCFA-receptor signaling. Our results suggested that FMT may improve NAFLD by reactivating SCFA-receptor signalling, which further supported the concept of the gut-liver axis in NAFLD pathogenesis.

For further verification that FMT influenced key protein expressions and fatty acid metabolism, the RT-PCR and WB results showed that FMT influenced the gene expression of SCFA receptors GPR41/43 and the protein expression of CIDEA, GPR135, p-AMPK/AMPK, and p-ACC1/ACC1. These proteins were closely related to hepatic steatosis development and lipid metabolism. Previous study reported that the activation of AMPK and the regulation of ACC1 were important for controlling lipid synthesis in the liver [50]. Our findings that FMT upregulated p-AMPK/AMPK and p-ACC1/ACC1 and downregulated CIDEA were consistent with these previous reports, indicating that FMT can modulate key proteins involved in lipid metabolism. In terms of fatty acid metabolism, untargeted metabolomics analysis indicated that FMT intervention modulated fatty acid levels in NAFLD mice. The downregulation of pro-lipogenic fatty acids such as palmitic acid and stearic acid after FMT treatment was consistent with the anti-lipogenic effects of FMT observed in the gene and protein expression analyses. However, the fact that docosahexaenoic acid (DHA) levels were not significantly upregulated after FMT, despite being downregulated in the model group, suggested that the anti-inflammatory pathways may not be fully restored. As previous studies have shown that DHA has anti-inflammatory properties, and its abnormal levels may be related to the incomplete recovery of the gut-liver axis in NAFLD [54, 55].

SCFAs ameliorated hepatic steatosis via the GPR135/AMPK-Cidea/ACC1 axis. In the in vitro LX2 model, SCFAs effectively reduced TG and TC accumulation and lipid droplet formation. The concentration-dependent upregulation of *GPR135* and *AMPK* mRNA expression and downregulation of *Cidea* and *ACC1* mRNA expression by SCFAs indicated that SCFAs may exert their therapeutic effects through dual regulatory mechanisms. A study by Zhou [56] also reported that SCFAs can regulate lipid metabolism through AMPK activation. However, our study is the first to propose that SCFAs exert their therapeutic effects through the GPR135/AMPK-Cidea/ACC1 axis this dual regulatory mechanism (SCFA receptor + downstream signaling) provides a more comprehensive explanation for SCFA’s anti-NAFLD effects, compared to the single AMPK pathway proposed in prior work. Our results further expanded on these findings by showing that SCFAs may also act through the regulation of SCFA receptors like GPR135, suggesting a more comprehensive mechanism for the treatment of NAFLD with SCFAs.

## Conclusion

The QKL was mostly composed of baicalin, cholic acid, porcine deoxycholic acid, and chlorogenic acid, among others. Administration of QKL to mice fed with an HFD effectively suppressed the progression of NAFLD in a dose-dependent manner, demonstrating effectiveness equivalent to that of MET. QKL significantly reduced hepatic cholesterol and triglyceride accumulation, along with their fat accumulation responses. Mice treated with QKL exhibited a decreased Firmicutes/Bacteroidota ratio and increased proliferation of advantageous gut microbiota, like *Akkermansia* and *Bacteroides*, which was accompanied by enhanced secretion of SCFAs (acetic, propionic, and butyric acids). Additionally, the integrity of the gut mucosal barrier was enhanced by the therapy, contributing to the overall maintenance of intestinal health. Moreover, QKL driven SCFAs regulated AMPK pathway through downregulating CIDEA and upregulating ACC1 to reduce liver lipid levels. Collectively, the process by which QKL anti-NAFLD may be realised by regulating gut microbiota to drive SCFAs to act on the AMPK/ACC1 signalling pathway. The application of QKL in NAFLD drug development held great significance. One limitation of QKL treatment for NAFLD lied in the species differences between mice and humans, highlighting the need for future research to validate these findings through well-designed clinical trials in human populations. Meanwhile, this study served as a valuable reference for future clinical trials and highlights the potential of QKL as a promising treatment for alleviating the global burden of NAFLD and obesity.

## Supplementary Information

Additional file 1.

Additional file 2.

Additional file 3.

Additional file 4.

## Data Availability

Data is provided within the manuscript or supplementary information files.

## Abbreviations

QKL: Qing-Kai-Ling
NAFLD: Nonalcoholic fatty liver disease
MET: Metformin
HFD: High-fat diet
AST: Aspartate aminotransferase
ALT: Alanine aminotransferase
TC: Total cholesterol
TG: Triglycerides
HDL-C: High-density lipoprotein cholesterol
LDL-C: Low-density lipoprotein cholesterol
ALT/AST: Alanine/aspartate aminotransferase
F/B: Firmicutes and Bacteroidetes ratio
GPR41/43: G protein-coupled receptor 41/43
AMPK: Adenosine 5′-monophosphate (amp)-activated protein kinase
ACC1: Acetyl-coa carboxylase 1
CIDEA: Cell death-inducing dffa-like effector A
SCFAs: Short-chain fatty acids
H&E: Hematoxylin-eosin
IHC: Immunohistochemical
FMT: Fecal microbiota transplantation
GC: Gas chromatography
GTT: Glucose tolerance test
ITT: Insulin tolerance test
WAT: White adipose tissue
UPLC-MS/MS: Ultra performance liquid chromatography/tandem mass spectrometry
PCA: Principal component analysis
