# Supplementary figures and images for: Qing-Kai-Ling oral liquid alleviates non-alcoholic fatty liver disease via remodeling gut microbiota and activating AMPK/ACC1 axis
Source: Chin Med. 2025 Oct 19;20:177. doi: 10.1186/s13020-025-01237-4 (PMC12536533; doi:10.1186/s13020-025-01237-4)

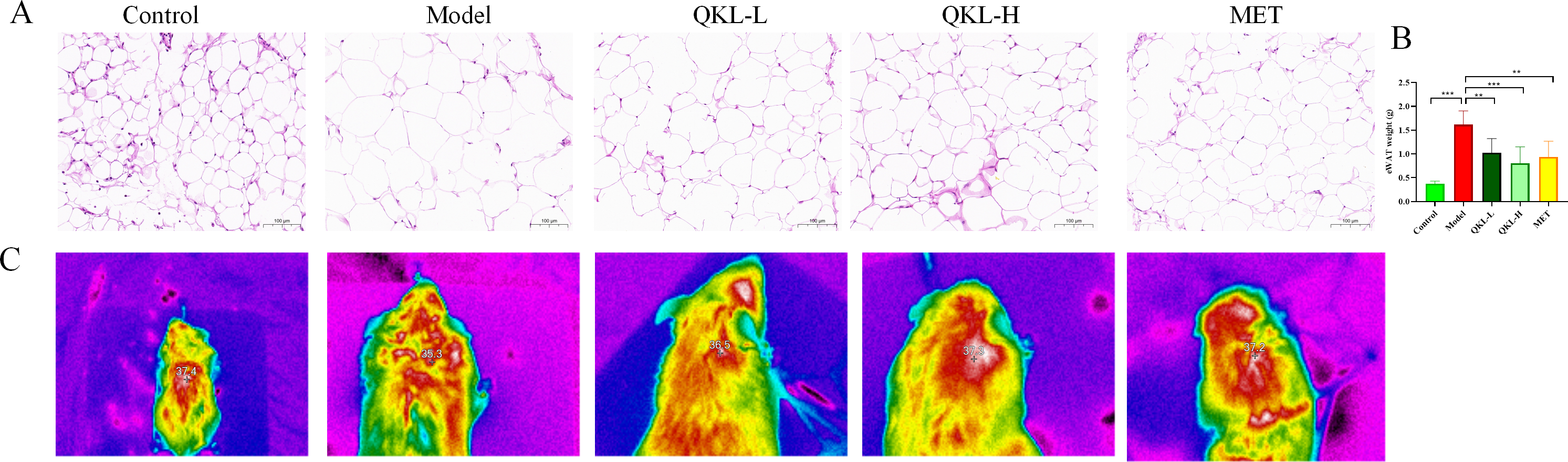

Supplement: Supplementary file 1 — Additional file 1. [file 13020_2025_1237_MOESM1_ESM.tif]

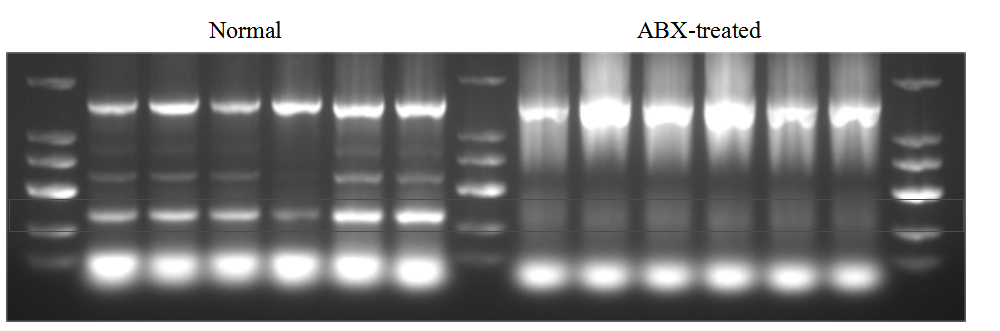

Supplement: Supplementary file 2 — Additional file 2. [file 13020_2025_1237_MOESM2_ESM.tif]
