# Supplementary material for: Qing-Kai-Ling oral liquid alleviates non-alcoholic fatty liver disease via remodeling gut microbiota and activating AMPK/ACC1 axis
Source: Chin Med. 2025 Oct 19;20:177. doi: 10.1186/s13020-025-01237-4 (PMC12536533; doi:10.1186/s13020-025-01237-4)
Supplement: Supplementary file 3 — Additional file 3. [file 13020_2025_1237_MOESM3_ESM.docx]

**Supplementary method 1**

Fecal DNA was extracted using the HiPure Stool DNA Kit and quantified via spectrophotometry. A 1% agarose gel was prepared in 1× TAE buffer (40 mM Tris-acetate, 1 mM EDTA, pH 8.0) and stained with GelRed nucleic acid dye. DNA samples (5 μL) were mixed with 6× loading buffer and loaded into wells alongside a DNA ladder (DL2000). Electrophoresis was performed at 100 V for 30 min, and DNA bands were visualized under UV light using a GelDoc system. Successful bacterial depletion was confirmed by the absence of distinct high-molecular-weight bands (>500 bp) in antibiotic-treated samples compared to controls.

**Supplementary method 2**

*Chemical composition identification of QKL*

The QKL oral liquid was diluted with 50% methanol, mixed with a scroll machine, and then passed through a 0.22 µm microporous filter membrane to obtain further filtrate for analysis. The HPLC-Q-TOF/MS technique was used to analyze the fecal samples after treatment with QKL oral liquid. UPLC adopts an Agilent TC-C18 column (4.6*250 mm, 5 mm). 0.1% formic acid water and acetonitrile (Darmstadt, Germany) constitute mobile phases A and B, respectively. The chromatographic separation was performed by gradient elution: 0 min: 10% B, 90% A; 60 min: 100% B, 0% A; 60.1 min: 10% B, 90% A; 70 min: 10% B, 90% A; The flow rate of the mobile phase is mainly 0.5 mL/min. The injection volume was 5 µL. The column temperature was set at 30°C. SCIEX X500R QTOF (AB SCIEX, USA) was used for mass spectrometry, including both negative and positive ion modes. Secondary mass spectrometry full scan. Positive and negative ionization voltage: ± 4500 V, spray gas: 50 psi, dry gas: 60 psi, sample cone-hole voltage: ± 80 V, ion source temperature: 450°C, impact gas: 7 cad, mass spectrum scanning range: 50-1000 Da, impact energy: 250 ± 10 ev. The data collection mode was automatic collection mode, analyzed by MZmine 2 software, and the possible molecular structure formula of the compound could be predicted by molecular formula prediction software.

**Supplementary method 3**

*Untargeted metabolomic analysis based on UPLC-Q/TOF-MS*

The chromatographic separation was achieved using an Acquity HSS T3 column (100 mm×2.1 mm, 1.8 μm) with a mobile phase consisting of 0.1% (v/v) formic acid in water (A) and 0.1% (v/v) formic acid in acetonitrile (B). The gradient elution proceeded as follows: 1% B from 0 to 2 min, increasing to 15% B from 2 to 3.5 min, 25% B from 3.5 to 7.5 min, 35% B from 7.5 to 9 min, 99% B from 9 to 11.5 min, maintaining 99% B from 11.5 to 17 min, decreasing back to 1% B from 17 to 17.1 min, and holding at 1% B from 17.1 to 20.1 min. The column and autosampler temperatures were kept at 40°C and 4°C, respectively.

The HPLC system was connected to a Xevo G2-S Q-TOF/MS (Waters) with a Q-Tra™ 4000 MS/MS system. The tandem mass spectrometer operated in both positive and negative electrospray ionization (ESI) modes with a mass-to-charge ratio acquisition range of 50 to 1000 m/z. Nitrogen (N_2_) was used as the drying gas at a flow rate of 1.5 L/min and a temperature of 350°C. The capillary voltages were set at 2500 V for positive ions and 2000 V for negative ions. The ion source temperature was 120°C with a cone voltage of 40 V, and collision energy ranged from 20 eV to 50 eV. To ensure precise mass measurement of each compound, leucine-enkephalin (m/z 556.2771) was used as the lock mass solution. These UPLC-Q/TOF-MS conditions were applied to fecal samples across all groups. Data acquisition and processing were conducted using Waters MassLynx™ V4.1 software.

**Supplement method 4**

*Gene expression profiling analysis and splicing analysis*

**①Sequencing Data Preprocessing**

Raw reads generated from sequencing were first filtered using fastp v0.18.0 to obtain high-quality clean reads, with filtering criteria as follows: (1) removing reads containing adapters; (2) removing reads with more than 10% unknown nucleotides (N); (3) removing low-quality reads with more than 50% bases of Q-value ≤20 . Next, the short reads alignment tool Bowtie2 v2.2.8 was used to map clean reads to the ribosome RNA (rRNA) database, and rRNA-mapped reads were discarded; the remaining clean reads were used for subsequent assembly and gene abundance calculation.

**②Library Preparation and Sequencing Platform**

The library preparation and RNA-seq transcriptome sequencing were carried out using the Illumina Novaseq X Plus platform (CA, USA) (a specific model of the Illumina® platform, consistent with the document’s recommended sequencing platform). Before library construction, fragmented mRNA (obtained via high-temperature treatment) was used as a template to synthesize first-strand cDNA with a reverse transcription enzyme mixture; during second-strand cDNA synthesis, end repair and A-tailing were completed simultaneously, followed by adapter ligation and target fragment selection using Hieff NGS® DNA Selection Beads.

**③Gene Expression Quantification**

An index of the reference genome was constructed, and paired-end clean reads were mapped to the reference genome using HISAT2 v2.1.0 (default parameters). The mapped reads of each sample were assembled via StringTie v1.3.1 (reference-based approach), and gene expression levels were quantified using RSEM software with the FPKM (Fragments Per Kilobase of transcript per Million mapped reads) metric (formula: *FPKM*=*NL*/103106*C*; C = number of fragments mapped to gene i; N = total number of fragments mapped to reference genes; L = number of bases on gene i) to eliminate the influence of gene length and sequencing data amount .

**④DEG Enrichment Analysis**

Enrichment profiling of differentially expressed genes (DEGs) in Gene Ontology (GO) categories (biological process, cellular component, molecular function) and Kyoto Encyclopedia of Genes and Genomes (KEGG) metabolic pathways was performed against the complete transcriptomic background. DEGs were first identified using DESeq2 software with thresholds set as false discovery rate (FDR) < 0.05 and absolute fold change ≥ 2.

For enrichment analysis: Significance was determined using FDR correction (instead of Bonferroni correction, consistent with the document’s standard for all enrichment analyses) with a threshold of FDR ≤ 0.05. Both GO and KEGG enrichment analyses adopted the hypergeometric test (calculation formula: *P*=1−∑*i*=0*m*−1​(*Nn*​) (*Mi*​)(*N*−*Mn*−*i*​)​; N = number of all genes with GO/KEGG annotation; n = number of DEGs in N; M = number of all genes annotated to a specific GO term/KEGG pathway; m = number of DEGs in M). The KEGG mapper tool via KOBAS-i (Beijing, China) was employed for pathway topology analysis, consistent with the document’s focus on KEGG pathway functional interpretation.

**Supplementary method 5**

*Cell culture and viability assay*

The human hepatic stellate cell line LX2 (Pricella Biotechnology company) was used for in vitro experiments. The cells were first retrieved from liquid nitrogen storage and subjected to thawing: rapidly thawed in a 37°C water bath, then transferred to a centrifuge tube containing pre-warmed high-glucose DMEM medium supplemented with 10% fetal bovine serum (FBS) and 1% penicillin-streptomycin. After centrifugation at 1,000 rpm for 5 min, the supernatant was discarded, and the cell pellet was resuspended in fresh complete medium, followed by seeding into cell culture dishes. The cells were incubated at 37°C in a humidified atmosphere with 5% CO_2_ for 2-3 days to allow recovery.

When the cell confluence reached approximately 80%, subculture was performed: the medium was aspirated, and the cells were rinsed twice with PBS. Then, 0.25% trypsin-EDTA solution was added to digest the cells at 37°C for 1-2 minutes until cell detachment was observed under a microscope. The digestion was terminated by adding complete medium, and the cell suspension was centrifuged at 1,000 rpm for 5 min. The cell pellet was resuspended, and the cells were seeded into new culture dishes at an appropriate density. This subculture process was repeated until the cells exhibited normal morphology (observed under a light microscope, characterized by typical stellate or polygonal shape, uniform size, and good adherence without signs of contamination) and stable growth status.

Subsequently, concentration screening for drug administration was conducted. The cells were seeded into 96-well plates at a density of 5×10^3^ cells per well and cultured overnight to allow adherence. Then, the test substances were added at gradient concentrations (100 μM, 200 μM, and 400 μM) and incubated with the cells for a specified period. After treatment, cell viability was assessed using the CCK-8 assay: 10 μL of CCK-8 reagent was added to each well, followed by incubation at 37°C for 2 hours. The absorbance at 450 nm was measured using a microplate reader, and the optimal non-toxic concentration (with cell viability maintained above 90% compared to the control group) was determined for subsequent experiments.
