# Supplementary material for: Qing-Kai-Ling oral liquid alleviates non-alcoholic fatty liver disease via remodeling gut microbiota and activating AMPK/ACC1 axis
Source: Chin Med. 2025 Oct 19;20:177. doi: 10.1186/s13020-025-01237-4 (PMC12536533; doi:10.1186/s13020-025-01237-4)
Supplement: Supplementary file 4 — Additional file 4. [file 13020_2025_1237_MOESM4_ESM.docx]

Table 1 Primer sequence for real-time polymerase chain reaction

| Gene | Primer sequence |
| --- | --- |
| *GPR41* |  |
| F | CACGGCCTACATCCTCATCT |
| R | TTGGTAGGTACCAGCGGAAG |
| *GPR43* |  |
| F | CTGGCGGAGCTACGTGCT |
| R | GGGGTCGATACAAGAGT |
| *GPR135* |  |
|  | GAGACCTTTGCCACCTTCACAGAG |
|  | CTTGGTCAAGGTCTTGGGTTGA |
| *ACC1* |  |
|  | TGCAGATCTGCTGCTGTGTG |
|  | TCACAGTTCAGCGGAAAGTG |
| *AMPK* |  |
| F | GCTTTCTGGGTGGACTCA AGT |
| R | GAGGGCAATCCGTCTTCATCC |
| *CIDEA* |  |
| F | GCCGAAGAGGTCGGGAATAG |
| R | TATCCACACGTGAACCT GCC |
| *GAPDH* |  |
| F | GGTGAAGGTCGGTGTGAAC |
| R | GAGTGGAGTCATAACTGGAAC |
